# Drivers of Inequality in Millennium Development Goal Progress: A Statistical Analysis

**DOI:** 10.1371/journal.pmed.1000241

**Published:** 2010-03-02

**Authors:** David Stuckler, Sanjay Basu, Martin McKee

**Affiliations:** 1 Oxford University, Department of Sociology, Oxford, United Kingdom; 2 London School of Hygiene & Tropical Medicine, Department of Public Health and Policy, London, United Kingdom; 3 Department of Medicine, University of California San Francisco, San Francisco, California, United States of America; 4 Division of General Internal Medicine, San Francisco General Hospital, San Francisco, California, United States of America; 5 European Observatory on Health Systems and Policies, Brussels, Belgium; University of Otago, New Zealand

## Abstract

**Background:**

Many low- and middle-income countries are not on track to reach the public health targets set out in the Millennium Development Goals (MDGs). We evaluated whether differential progress towards health MDGs was associated with economic development, public health funding (both overall and as percentage of available domestic funds), or health system infrastructure. We also examined the impact of joint epidemics of HIV/AIDS and noncommunicable diseases (NCDs), which may limit the ability of households to address child mortality and increase risks of infectious diseases.

**Methods and Findings:**

We calculated each country's distance from its MDG goals for HIV/AIDS, tuberculosis, and infant and child mortality targets for the year 2005 using the United Nations MDG database for 227 countries from 1990 to the present. We studied the association of economic development (gross domestic product [GDP] per capita in purchasing-power-parity), the relative priority placed on health (health spending as a percentage of GDP), real health spending (health system expenditures in purchasing-power-parity), HIV/AIDS burden (prevalence rates among ages 15–49 y), and NCD burden (age-standardised chronic disease mortality rates), with measures of distance from attainment of health MDGs. To avoid spurious correlations that may exist simply because countries with high disease burdens would be expected to have low MDG progress, and to adjust for potential confounding arising from differences in countries' initial disease burdens, we analysed the variations in rates of *change* in MDG progress versus expected rates for each country. While economic development, health priority, health spending, and health infrastructure did not explain more than one-fifth of the differences in progress to health MDGs among countries, burdens of HIV and NCDs explained more than half of between-country inequalities in child mortality progress (*R* ^2^-infant mortality  = 0.57, *R* ^2^-under 5 mortality  = 0.54). HIV/AIDS and NCD burdens were also the strongest correlates of unequal progress towards tuberculosis goals (*R* ^2^ = 0.57), with NCDs having an effect independent of HIV/AIDS, consistent with micro-level studies of the influence of tobacco and diabetes on tuberculosis risks. Even after correcting for health system variables, initial child mortality, and tuberculosis diseases, we found that lower burdens of HIV/AIDS and NCDs were associated with much greater progress towards attainment of child mortality and tuberculosis MDGs than were gains in GDP. An estimated 1% lower HIV prevalence or 10% lower mortality rate from NCDs would have a similar impact on progress towards the tuberculosis MDG as an 80% or greater rise in GDP, corresponding to at least a decade of economic growth in low-income countries.

**Conclusions:**

Unequal progress in health MDGs in low-income countries appears significantly related to burdens of HIV and NCDs in a population, after correcting for potentially confounding socioeconomic, disease burden, political, and health system variables. The common separation between NCDs, child mortality, and infectious syndromes among development programs may obscure interrelationships of illness affecting those living in poor households—whether economic (e.g., as money spent on tobacco is lost from child health expenditures) or biological (e.g., as diabetes or HIV enhance the risk of tuberculosis).

*Please see later in the article for the Editors' Summary*

## Abstract 2

David Stuckler and colleagues examine the impact of the HIV and noncommunicable disease epidemics on low-income countries' progress toward the Millennium Development Goals for health.

## Editors' Summary

**Background:**

In 2000, 189 countries adopted the United Nations (UN) Millennium Declaration, which commits the world to the eradication of extreme poverty by 2015. The Declaration lists eight Millennium Development Goals (MDGs), 21 quantifiable targets, and 60 indicators of progress. So, for example, MDG 4 aims to reduce child mortality (deaths). The target for this goal is to reduce the number of children who die each year before they are five years old (the under-five mortality rate) to two-thirds of its 1990 value by 2015. Indicators of progress toward this goal include the under-five mortality rate and the infant mortality rate. Because poverty and ill health are inextricably linked—ill health limits the ability of individuals and nations to improve their economic status, and poverty contributes to the development of many illnesses—two other MDGs also tackle public health issues. MDG 5 sets a target of reducing maternal mortality by three-quarters of its 1990 level by 2015. MDG 6 aims to halt and begin to reverse the spread of HIV/AIDS, malaria, and other major diseases such as tuberculosis by 2015.

**Why Was This Study Done?:**

Although progress has been made toward achieving the MDGs, few if any of the targets are likely to be met by 2015. Worryingly, low-income countries are falling furthest behind their MDG targets. For example, although child mortality has been declining globally, in many poor countries there has been little or no progress. What is the explanation for this and other inequalities in progress toward the health MDGs? Some countries may simply lack the financial resources needed to combat epidemics or may allocate only a low proportion of their gross domestic product (GDP) to health. Alternatively, money allocated to health may not always reach the people who need it most because of an inadequate health infrastructure. Finally, coexisting epidemics may be hindering progress toward the MDG health targets. Thus, the spread of HIV/AIDS may be hindering attempts to limit the spread of tuberculosis because HIV infection increases the risk of active tuberculosis, and ongoing epidemics of diabetes and other noncommunicable diseases (NCDs) may be affecting the attainment of health MDGs by diverting scarce resources. In this study, the researchers investigate whether any of these possibilities is driving the inequalities in MDG progress.

**What Did the Researchers Do and Find?:**

The researchers calculated how far 227 countries were from their MDG targets for HIV, tuberculosis, and infant and child mortality in 2005 using information collected by the UN. They then used statistical methods to study the relationship between this distance and economic development (GDP per person), health spending as a proportion of GDP (health priority), actual health system expenditures, health infrastructure, HIV burden, and NCD burden in each country. Economic development, health priority, health spending, and health infrastructure explained no more than one-fifth of the inequalities in progress toward health MDGs. By contrast, the HIV and NCD burdens explained more than half of inequalities in child mortality progress and were strongly associated with unequal progress toward tuberculosis goals. Furthermore, the researchers calculated that a 1% reduction in the number of people infected with HIV or a 10% reduction in rate of deaths from NCDs in a population would have a similar impact on progress toward the tuberculosis MDG target as a rise in GDP corresponding to at least a decade of growth in low-income countries.

**What Do These Findings Mean?:**

These findings are limited by the quality of the available data on health indicators in low-income countries and, because the researchers used country-wide data, their findings only reveal possible drivers of inequalities in progress toward MDGs in whole countries and may mask drivers of within-country inequalities. Nevertheless, as one of the first attempts to analyze the determinants of global inequalities in progress toward the health MDGs, these findings have important implications for global health policy. Most importantly, the finding that unequal progress is related to the burdens of HIV and NCDs in populations suggests that programs designed to achieve health MDGs must consider all the diseases and factors that can trap households in vicious cycles of illness and poverty, especially since the achievement of feasible reductions in NCDs in low-income countries could greatly enhance progress towards health MDGs.

**Additional Information:**

Please access these Web sites via the online version of this summary at http://dx.doi.org/10.1371/journal.pmed.1000241.

- The United Nations Millennium Development Goals website provides detailed information about the Millennium Declaration, the MDGs, their targets and their indicators
- The Millennium Development Goals Report 2009 and its progress chart provide an up-to-date assessment of progress towards the MDGs
- The World Health Organization provides information about poverty and health and health and development

## Introduction

Remarkable efforts have been made towards meeting the health MDGs over the past 8 years (Box 1), yet many of the poorest countries are falling behind. As noted in the Report of the Secretariat to the World Health Assembly, “At the mid-point in the countdown to 2015, the target date set by the United Nations Millennium Declaration, there are several examples of success. However, great inequalities still exist within and between countries, and current trends suggest that many low-income countries will not reach the Millennium Development Goal targets” [1].

**Box 1. Health Millennium Development Goals, Targets, and Indicators**

In 2001, 192 United Nations member states agreed upon three Millennium Development Goals (MDGs) to reduce child mortality rates by two-thirds (MDG #4), maternal mortality ratios by three-quarters (MDG #5), and halt and reverse the spread of HIV, tuberculosis, and malaria by 2015 (MDG #6). ***Note***: *Health outcome indicators available for study on a longitudinal basis are italicised.*

**Goal 4: Reduce child mortality**

- Target 4.A: Reduce by two-thirds, between 1990 and 2015, the under-five mortality rate
  - *4.1 Under-five mortality rate*
*4.2 Infant mortality rate*
  - 4.3 Proportion of 1 year-old children immunised against measles

**Goal 5: Improve maternal health**

- Target 5.A: Reduce by three quarters, between 1990 and 2015, the maternal mortality ratio
  - 5.1 Maternal mortality ratio
  - 5.2 Proportion of births attended by skilled health personnel
- Target 5.B: Achieve, by 2015, universal access to reproductive health
  - 5.1 Contraceptive prevalence rate
  - 5.2 Adolescent birth rate
  - 5.3 Antenatal care coverage (at least one visit and at least four visits)
  - 5.4 Unmet need for family planning

**Goal 6: Combat HIV/AIDS, malaria and other diseases**

- Target 6.A: Have halted by 2015 and begun to reverse the spread of HIV/AIDS
  - *6.1 HIV prevalence among population aged 15–24 years*
  - 6.2 Condom use at last high-risk sex
  - 6.3 Proportion of population aged 15–24 years with comprehensive correct knowledge of HIV/AIDS
  - 6.4 Ratio of school attendance of orphans to school attendance of non-orphans aged 10–14 years
- Target 6.B: Achieve, by 2010, universal access to treatment for HIV/AIDS for all those who need it
  - 6.5 Proportion of population with advanced HIV infection with access to antiretroviral drugs
- Target 6.C: Have halted by 2015 and begun to reverse the incidence of malaria and other major diseases
  - 6.6 Incidence and death rates associated with malaria
  - 6.7 Proportion of children under 5 sleeping under insecticide-treated bed nets
  - 6.8 Proportion of children under 5 with fever who are treated with appropriate anti-malarial drugs
  - *6.9 Incidence, prevalence and death rates associated with tuberculosis*
  - 6.10 Proportion of tuberculosis cases detected and cured under directly observed treatment short course

What explains these inequalities in progress toward the health MDGs [2]? Slow progress in low-income countries cannot simply be explained by their public health MDG targets being more challenging. Reducing child mortality by two-thirds (MDG 4.1) or maternal mortality by three-quarters (MDG 5.1) may be more difficult when death rates are already low, as in rich countries. One possibility is that countries simply lack the financial resources needed to combat epidemics (i.e., low gross domestic product [GDP] per capita). Even when funds are available, they may be allocated to other forms of social spending, military expenditure, or reserves, rather than to health (i.e., low health spending for each dollar of GDP). A third possibility is that when funds enter the health system, inadequate health infrastructure—such as a lack of doctors, pharmaceuticals, or hospitals—prevent these allocations from reaching those who need them most (i.e., low absorptive capacity for spending).

However, another contributing factor could be coexisting epidemics. For example, the spread of HIV has slowed progress towards achievement of the tuberculosis goals by directly increasing the risk of active tuberculosis disease and subsequent death [3]. There are also multiple pathways by which higher adult HIV prevalence might increase child mortality (beyond the obvious mother to child transmission), estimated to account for a significant fraction of deaths in sub-Saharan Africa [4]. These include direct effects on the welfare of children (reduced earning capacity of parents, cost of treatment, impoverishment of orphans) and indirect mechanisms involving depletion of resources (illness and death of health workers and teachers, diversion of resources from child health care) [5]–[7].

In recent years, attention has turned to the contributions made by long-term noncommunicable conditions to the overall burden of disease in poor countries [8]. These include cardiovascular disorders, chronic obstructive pulmonary disease, diabetes, and common cancers as well as disabling mental illness and injuries. It is increasingly clear that the greatest burden of NCDs is among the poor [9]–[13]. Their plight is not simply an inevitable consequence of aging, as is sometimes argued [14]. Instead, traditional diets are giving way to cheaper, unhealthy alternatives (the so-called “nutrition transition”) [15], transnational tobacco companies aggressively market their products in the developing world, and urbanization and associated changes in employment have reduced physical activity [16]–[18]. Crucially, in the present context, the nature of the transition means that many families face a double burden of what are sometimes referred to as diseases of affluence and poverty [19]. For example, 44% of families with an undernourished member in Brazil also had an overweight member, with high prevalence also observed in China (23%) and Russia (58%) [20]. A survey of very-low-income populations in Maceio, Brazil found that 30% of all families had both an underweight and overweight–obese member living under the same roof [21].

As with HIV/AIDS, NCDs and their risk factors can impact adversely on attainment of the health MDGs (Table 1). In some cases this is due to the biological consequences of NCDs and their risk factors. For example, in the case of tuberculosis, WHO notes that “Risk factors that seem to be of importance at the population level include poor living and working conditions associated with high risk of TB transmission, and factors that impair the host's defence against TB infection and disease, such as HIV infection, malnutrition, *smoking, diabetes, alcohol abuse, and indoor air pollution*.” (emphases added) [22]. Although the increased relative risk of tuberculosis associated with smoking and diabetes is less than for HIV infection, in some populations the higher prevalence of these factors leads to a greater population attributable risk. A recent study by Dye and colleagues in India estimated that diabetes accounts for 20% of smear-positive tuberculosis incidence, with the higher prevalence of diabetes in urban areas explaining one-fifth of the gap in smear-positive disease between urban and rural areas [23]; other studies in Latin America have attributed as much as one-quarter of pulmonary tuberculosis incidence to diabetes and, in India, over half of tuberculosis mortality to tobacco [11],[24],[25]. Among children, exposure to second-hand tobacco smoke and smoke-producing stoves increases risks of respiratory infections (one of the leading causes of death in the very poorest children) and sudden infant death.

**Table 1:** Selected Effects of NCDs and injuries and their risk factors on health MDGs.

Health MDG	Type of Pathway	Effect of NCD and NCD risk factors on health MDG
MDG #4. Reduce Child Mortality	Biological	Tobacco increases probability of low birthweight [61],[64]
	Social	Alcohol, tobacco and out-of-pocket long-term chronic disease care household expenditures displace spending on nutrition (up to 500 calories per child per day) [26],[28],[65]
MDG #5. Improve Maternal Health	Biological	Tobacco, obesity and diabetes create high-risk childbirth conditions [66]
MDG #6. Combat HIV/AIDS, malaria and other diseases [including tuberculosis]	Biological	Tobacco increases risk of tuberculosis by about 2-fold [11],[25]
	Biological	Diabetes increases risk of tuberculosis and MDR by about 3-fold; estimated to be attributable for 10% of TB in India and China and 15% globally [23],[67]
	Biological	Tobacco increase risks for HIV infection [Furber AS, Maheswaran R, Newell JN, Carroll C (2007) Is smoking tobacco an independent risk factor for HIV infection and progression to AIDS? A systemic review. Sex Transm Infect 83: 41-46.]

*Notes*: See Text S6 for more details.

However, expenditures on the risk factors for NCDs and the management of these disorders can also impact adversely on the financial well-being of families, placing them at risk in relation to the conditions included in the health MDGs. Thus, tobacco expenditures in Bangladesh have exceeded spending on health, education, and clothing by a factor of five [26]; this spending was found to have been displaced from nutrition and health care, where it would have added as much as 500 calories per day to the diets of otherwise potentially undernourished children [27]. Treatment for diabetes costs 15%–25% of incomes in households with an affected person in India [28], 25% of the minimum wage (20 times the per capita health expenditure) in Tanzania [29], 6–12 months' wages (US$160 per year) in Bangladesh [30], and roughly US$550 per person in Latin America (more than average per capita health expenditure) [31], while health care for smoking-related diseases accounts for 0.48% of GDP in Thailand [32]. Research from countries as diverse as Burkina Faso [33] and Thailand [34] find that the presence of a chronic illness in a family is one of the most important determinants of whether the household will incur catastrophic health expenditure.

New insights on the importance of NCDs are being obtained from longitudinal studies that track health expenditure in families, rather than previous facility-based studies of utilization. Thus, in South Africa a household survey found that 74% of reported health problems were “chronic,” 48% of which had received no treatment in the previous month. In a linked follow-up of households, among subjects with chronic illness, only 62% had an allopathic diagnosis and only 35% were receiving regular treatment [35]. A study in India found that chronic diseases represented 17.7% of illnesses but 32% of costs. Although hospitalizations were the single most costly component on average, they accounted for only 11% of total costs, compared to drugs, accounting for 49% of total costs [36].

Finally, the onset of an NCD also appears to restrict earning potential [37]–[41] and undermine a family's ability to provide for children. High background rates of chronic NCD morbidity and mortality among adults results in losses of adult care providers, and disability among adults prevents efforts to secure child health, or to obtain diagnoses and complete treatment for infectious diseases.

Table 1 further summarises the evidence of how high burdens of NCDs impede progress to health MDGs.

In this paper, we compare several of the leading explanations of slow progress towards health MDGs, including the effects of low economic development, a lack of health prioritisation by governments, low absolute health spending, and scarce health infrastructure. While some recent studies identify barriers to achieving progress towards MDGs in individual countries or regions (especially sub-Saharan Africa) or for individual diseases [42],[43], there remains a need to test the relationships across health MDGs and low-, middle-, and high-income countries. For what we believe is the first time, we also test the hypothesis that coexisting epidemics of HIV/AIDS and NCDs impede progress towards the health MDGs, after controlling for the potential economic and health system barriers to achieving the health MDGs.

### Data and Methods

We extracted data on MDG indicators from the United Nations Millennium Development Goal Indicator Database 2009 edition for 227 countries spanning 1990 to 2008 [44]. Complete time series data related to some health MDGs in low income countries were missing from the UN repository (Text S1). This was a particular problem with maternal mortality and malaria. Much of the information on maternal mortality in low-income countries derives from the sisterhood method, which is designed to yield a lifetime risk [45] and, while data on the prevalence of malaria are now much more widely available, historical data are very limited [46]. Hence we limited the analysis to the indicators available for evaluating rates of progress toward the MDG target, which corresponded to child health, tuberculosis, and HIV. Fortunately, these are also the health MDGs where we hypothesise that an association with NCD burden might be expected. In Box 1, we highlight the MDG indicators used in the assessment.

We calculated the change in the appropriate variable that would be required by 2005 to place each country on target to achieve each MDG. For example, MDG 4, which aims “to reduce infant and child mortality by two-thirds by 2015,” would require a 40% reduction in infant and child mortality rates by 2005 assuming a uniform (linear) rate of progress. We note that this linear transformation of the outcome variable is statistically equivalent to an alternative approach of first modelling the real values, then offsetting by the baseline mortality data; however, to ensure consistency with our hypothesis, we present the transformed results. The health MDG targets are set out at http://www.un.org/millenniumgoals/.

To measure “unmet MDG progress,” we then divided the actual change in the variable achieved by 2005 by the target for that year: 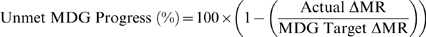
(1)


Using the above example, the resulting value would be 25% for a country achieving a 30% reduction in infant and child mortality rates (100×[(1−(30/40)]) or, put more simply, would be 25% adrift from where it should be by 2005. Countries which exceeded the MDG target were coded as negative unmet progress (although the results were unchanged when the score was truncated at zero unmet progress). For countries which moved in reverse, or experienced rises in mortality rates (e.g. a total of 13 countries for infant mortality), it was possible to have greater than 100% unmet MDG progress.

Data on MDG 5, “to reduce maternal mortality by two-thirds,” were available only on a comparative basis for 2005. MDG 6, “to halt and reverse the incidence, prevalence and mortality of HIV, malaria and other diseases [including tuberculosis],” was coded as a dichotomous variable. Countries which experienced rises in tuberculosis mortality and HIV prevalence rates were assigned the value 1, denoting unmet progress, whereas those which had no change or reduction were assigned 0. This enabled comparisons of progress in both MDG 4 and 6 based on the probability of (or the percentage of) MDG success.

We used the World Bank's methods [47] to designate countries as low-, middle-, and high-income, based not on their yearly GDP per capita but on their average GDP per capita from 1990 to 2005. This avoids classifying countries with successful growth strategies as higher-income, or poor economic performers as lower-income, which could potentially bias a statistical analysis of mortality changes among them.

To study the determinants of progress to the MDGs, we modelled the MDG indicators as:
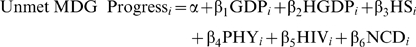
(2)


where *i* is country. GDP is GDP per capita in purchasing-power-parity from the Penn World Tables version 6.2 for the year 2003 (because there were data for 92 additional countries in this version of the tables than in more recent editions); HGDP is health spending as a percentage of GDP from the WHO Statistical Information Database for the year 2005; HS is health spending per capita in purchasing-power-parity and PHY is the number of physicians per 10,000 population, both from the WHO Statistical Information Database for the year 2005 [48]; HIV is HIV prevalence among ages 15–49 y, the HIV indicator available from the UN MDG database for the years 2006/2007; and NCD is the WHO Global Burden of Disease estimates of NCD mortality rates for the year 2004 (the most recent year available from WHO [49]; this includes WHO classification scheme Group 2 causes, age-standardised to the WHO World Standard Population to adjust for potential errors arising from differences in the population age-structure [50], given that NCDs contribute a greater share of deaths at older ages) [48]. Log transformations were applied to GDP and NCD data to adjust for positive skew and aid model fitting. Note that we analysed the variance in the rates of change in MDG progress relative to baseline rates of change, correcting for spurious correlations that may exist simply because countries with high disease burdens would be expected to have low MDG progress. We specifically controlled for the initial burden of disease estimates and rates of change in those estimates among countries when performing our regressions by incorporating expected mortality rates in the calculation of unmet MDG progress. Huber/White sandwich estimators of standard errors are presented for consistency in the presence of potential heteroskedasticity. To facilitate the interpretation of the models, we have transformed estimated coefficients into elasticities and interpreted the effect sizes based on units to improve clarity and comparability. Data were analysed using STATA 10.1. All data are available upon request from the authors.

## Results

### Inequalities in Progress to Health MDGs

**Figure 1:**
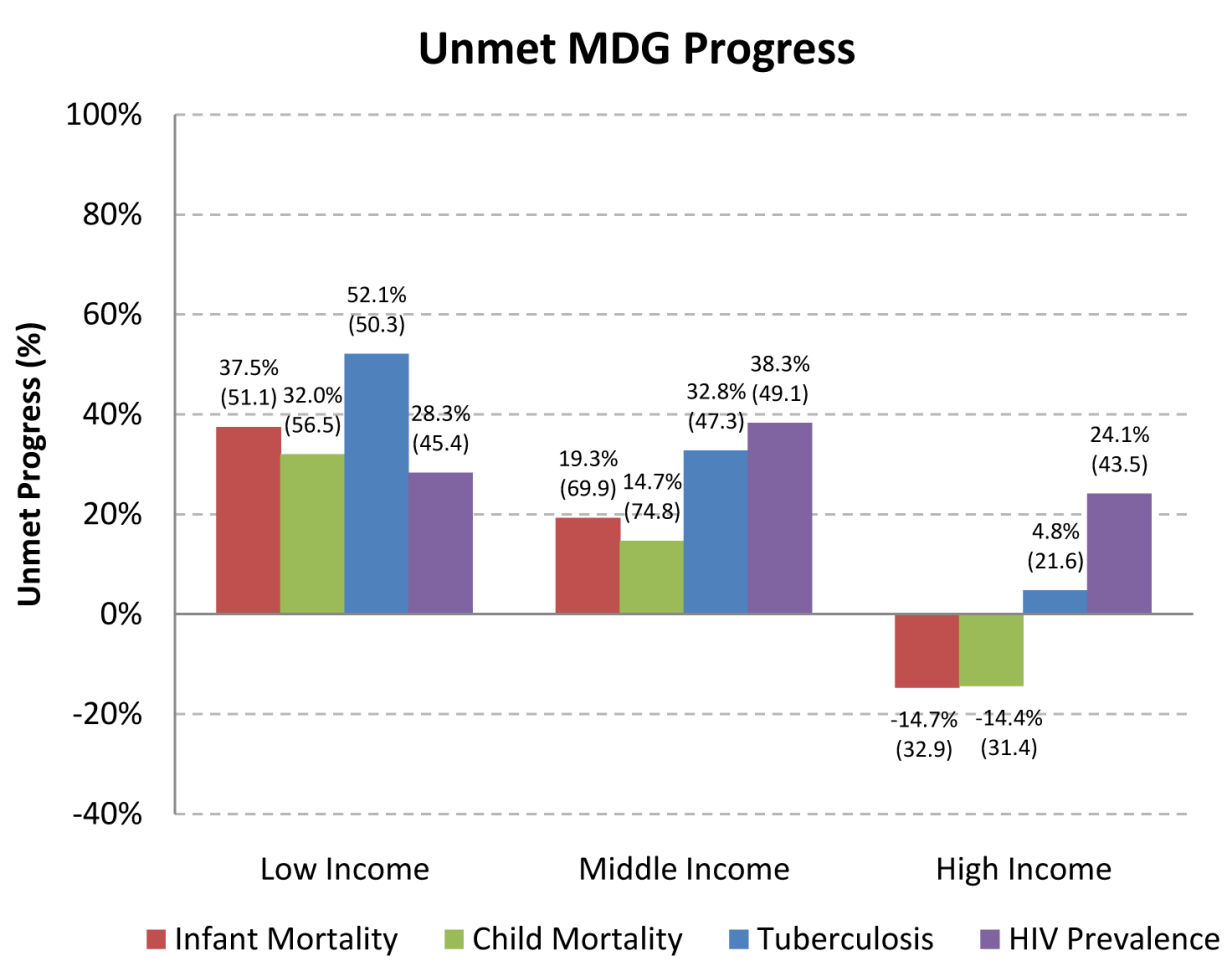
Unmet progress towards Millennium Development Goals, by income group. Notes: Authors' calculations. Unmet MDG Progress is calculated in percentage terms as 100 * [1–(Actual ΔMR/Expected ΔMR)], for years 1990 and 2005 for infant, child, and tuberculosis mortality; 2001 and 2007 for HIV prevalence. Scores include negative values (i.e., greater than 100% progress). Source of data: Millennium Development Goals Indicators, available at http://mdgs.un.org/unsd/mdg/Default.aspx. MDG #4 aims to reduce infant and child mortality by two-thirds by 2015. MDG #6 aims to halt and reverse the incidence, prevalence and mortality of HIV, malaria and other diseases, including tuberculosis. Box 1 further defines MDG targets. Income groups categorized based on the World Bank Atlas method based on their average GDP per capita from 1990 to 2005. For Box plots see Text S8

Figure 1 compares progress toward health MDGs among low-, middle-, and high-income countries and by World Bank region. Out of 71 low-income countries for which data are available, rising death rates have been recorded in 37 countries for tuberculosis since 1990 (MDG 6.9); in seven and nine countries for infant and child mortality rates, respectively, since 1990 (MDG 4.1 and 4.2); and in 17 countries for HIV prevalence (MDG 6.1) from 2001 to 2007 (the period for which data are available). Compared to high- and middle-income countries, low-income countries are about one-quarter less likely to be on pace to reach the HIV and TB targets set out in MDG 6 (23.7%, 95% CI 7.8%–39.4%) and the child mortality targets in MDG 4 (27.6%, 95% CI 10.5%–44.7%).

**Figure 2:**
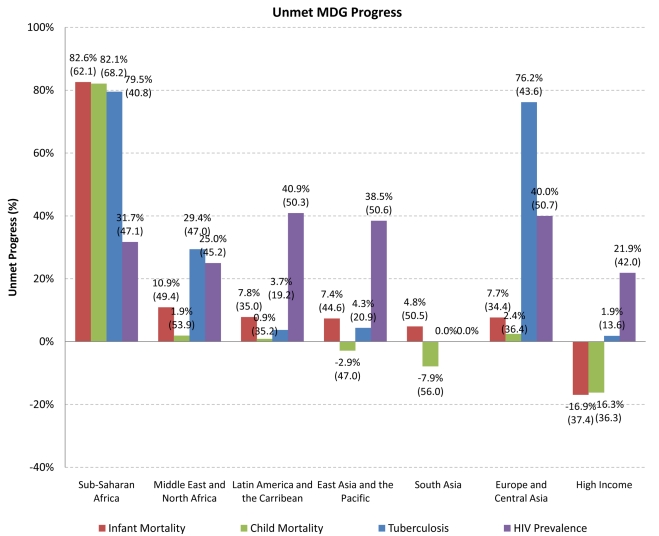
Unmet progress towards Millennium Development Goals, by geographic region. *Notes*: Authors' calculations. Unmet MDG Progress is calculated in percentage terms as 100 * [1–(Actual ΔMR/Expected ΔMR)], for years 1990 and 2005 for infant, child, and tuberculosis mortality; 2001 and 2007 for HIV prevalence. Scores include negative values (i.e., greater than 100% progress). Source of data: Millennium Development Goals Indicators, available at http://mdgs.un.org/unsd/mdg/Default.aspx. MDG #4 aims to reduce infant and child mortality by two-thirds by 2015. MDG #6 aims to halt and reverse the incidence, prevalence and mortality of HIV, malaria and other diseases, including tuberculosis. Box 1 further defines MDG targets. Zero percent denotes complete progress. Geographic classification based on World Bank geographic categories for the year 2009: http://web.worldbank.org/WBSITE/EXTERNAL/DATASTATISTICS/0,,contentMDK:20420458~menuPK:64133156~pagePK:64133150~piPK:64133175~theSitePK:239419,00.html

As shown in Figure 2, sub-Saharan Africa had the slowest progress (82.1% unmet progress in child mortality, 79.5% in tuberculosis mortality, and 31.7% in HIV prevalence), followed by Europe and Central Asia (2.4% unmet progress in child mortality, 76.2% in tuberculosis mortality, and 40.0% in HIV prevalence).

At current rates of progress, fewer than half of low-income countries will achieve the 2015 targets set for HIV prevalence or infant, child, and tuberculosis mortality rates. Inequalities between the global north and south have been rising, as progress towards MDGs has not only been much slower in the south, but has moved into reverse for a substantial number of the lowest-income countries, as indicated by the >100% unmet progress (Text S2).

### Economic Development, Health Systems, and Health MDGs

We now examine each of the possible explanations for inequalities in progress towards the MDGs postulated earlier.

The first possibility is that poor countries simply lack the financial resources to tackle their public health goals. Table 2 shows the results of a statistical model relating the level of earnings (GDP per capita in purchasing-power-parity) to unmet MDG progress. The model reveals that scaling up current forms of economic development is not sufficient for success [11]. Each 10% higher GDP per capita was associated with 1.80% greater progress towards the infant mortality targets (95% CI 1.25%–2.35%), 1.64% greater progress towards the under-5 mortality target (95% CI 1.05%–2.24%), and 1.64% greater progress toward the tuberculosis mortality targets (95% CI 1.13%–2.14%). However, less than one-sixth of unmet progress in combating infant, child, and tuberculosis mortality, and almost none of progress in combating HIV, could be attributed to differences in economic development in this regression analysis (see also Text S2 for representative values).

**Table 2:** Associations of GDP per capita with percentage of unmet progress towards health-related MDGs.

Covariate	Quantity of Unmet MDG Progress			
	Infant Mortality Rates	Child Mortality Rates	Tuberculosis Mortality Rates	HIV Prevalence
10% higher GDP per capita	−1.80%*** [−2.35 to −1.25]	−1.64%*** [−2.24 to −1.05]	−1.64%*** [−2.14 to −1.13]	0.039% [−0.62 to 0.70]
Number of countries	164	164	166	132
*R* ^2^	0.130	0.098	0.168	<0.001

*Notes*: Results presented from four separate regression models. Constant estimated but not reported. 95% confidence intervals based on heteroskedasticity robust standard errors in parentheses. Unmet MDG Progress is calculated in percentage terms as 100×[1−(Actual ΔMR/Expected ΔMR)]. Multiplying each coefficient by 40% transforms the coefficient to describe the associations with the percentage change in each outcome. Progress towards reducing infant mortality rates and child mortality rates reflects MDG 4.1 and 4.2 and modelled using a linear standard regression model. Progress towards halting or reversing tuberculosis mortality rates reflects MDG 6.9, and modelled using a linear probability model. Progress towards halting or reversing HIV prevalence reflects MDG 6.1, and modelled using a linear probability model. Data are from UN Millennium Development Goals Indicators 2008 edition.

*p<0.05.

**p<0.01.

***p<0.001.

Another possibility is that overall financial resources are theoretically sufficient but not being used to strengthen health systems. We next included health spending as a percentage of GDP as a measure of governmental priority given to health. While each 1% higher health spending as a percentage of GDP was associated with faster progress to the MDGs, the effects were not statistically significant except for declines in HIV prevalence (each percentage increase in spending/GDP changes HIV prevalence by −4.50% (95% CI−8.39% to−0.61%) (Table 3).

**Table 3:** Associations of GDP per capita and health spending/GDP with percentage of unmet progress towards health-related MDGs.

Covariate	Quantity of Unmet MDG Progress			
	Infant Mortality Rates	Child Mortality Rates	Tuberculosis Mortality Rates	HIV Prevalence
10% higher GDP per capita	−1.76%*** [−2.42 to −1.11]	−1.63%*** [−2.33 to −0.93]	−1.66%*** [−2.23 to −1.08]	0.43% [−0.32 to 1.18]
Health Spending as percentage of GDP	−0.67% [−4.49 to 3.15]	−0.41% [−4.48 to 3.67]	−0.45% [−3.15 to 2.26]	−4.50%* [−8.39 to −0.61]
Number of Countries	163	163	163	131
*R* ^2^	0.131	0.098	0.174	0.035

*Notes*: Results presented from four separate regression models. Constant estimated but not reported. 95% confidence intervals based on heteroskedasticity robust standard errors in parentheses. Unmet MDG Progress is calculated in percentage terms as 100×[1−(Actual ΔMR/Expected ΔMR)]. Multiplying each coefficient by 40% transforms the coefficient to describe the associations with the percentage change in each outcome. Progress towards reducing infant mortality rates and child mortality rates reflects MDG 4.1 and 4.2 and modelled using a linear standard regression model. Progress towards halting or reversing tuberculosis mortality rates reflects MDG 6.9, and modelled using a linear probability model. Progress towards halting or reversing HIV prevalence reflects MDG 6.1, and modelled using a linear probability model. Data are from UN Millennium Development Goals Indicators 2008 edition.

*p<0.05.

**p<0.01.

***p<0.001.

But perhaps financial resources or the proportion of these resources apportioned to health are not as relevant as the absolute number of dollars devoted to health per capita or the physical capacity in the health care system. Hence, we next evaluated the effects of real health spending and physicians per capita on health MDG progress. We found no effect of greater health spending (Table 4), but when we added physicians per capita to the model (Table 5) we found that each 1 additional doctor per 10,000 population was strongly associated with greater progress towards reducing progress toward infant mortality targets (1.43%, 95% CI 0.38%–2.49%) and under-5 mortality rates (1.44%, 0.31%–2.57%). After including physicians per capita in the model, GDP per capita also no longer had a significant association with progress on child mortality (*p* = 0.3721), which is consistent with a potential pathway linking GDP to greater human capital and subsequent greater progress towards the MDGs. However, we found that greater numbers of physicians per capita was associated with slower progress in tuberculosis mortality rates (−0.98%, 95% CI−0.24% to −1.73%), which could indicate bias due to better surveillance or, possibly, the contribution of poorly regulated health care provision to the emergence of drug resistance [51],[52]. The full model—including real GDP per capita, health as a percentage of GDP, real health spending per capita, and physicians per capita—explained roughly one-fifth of country inequalities, which leaves a sizable residual to be accounted for.

**Table 4:** Associations of GDP per capita, health spending/GDP, and health spending with percentage of unmet progress toward health MDGs.

Covariate	Quantity of Unmet MDG Progress			
	Infant Mortality Rates	Child Mortality Rates	Tuberculosis Mortality Rates	HIV Prevalence
10% higher GDP per capita	−1.55%** [−2.46 to −0.64]	−1.41%** [−2.37 to −0.45]	−1.69%*** [−2.42 to −0.96]	0.78% [−0.22 to 1.78]
1% higher Health Spending as percentage of GDP	0.11% [−4.68 to 4.89]	0.37% [−4.76 to 5.49]	−0.56% [−3.91 to 2.78]	−3.29% [−7.35 to 0.77]
$10 higher Health Spending per capita (PPP)	−0.054% [−0.18 to 0.075]	−0.054% [−0.19 to 0.079]	0.0082% [−0.083 to 0.099]	−0.084% [−0.20 to 0.036]
Number of Countries	163	163	163	131
*R* ^2^	0.133	0.100	0.174	0.045

*Notes*: Results presented from four separate regression models. Constant estimated but not reported. 95% confidence intervals based on heteroskedasticity robust standard errors in parentheses. Unmet MDG Progress is calculated in percentage terms as 100×[1–(Actual ΔMR/Expected ΔMR)]. Multiplying each coefficient by 40% transforms the coefficient to describe the associations with the percentage change in each outcome. Progress towards reducing infant mortality rates and child mortality rates reflects MDG 4.1 and 4.2 and modelled using a linear standard regression model. Progress towards halting or reversing tuberculosis mortality rates reflects MDG 6.9, and modelled using a linear probability model. Progress towards halting or reversing HIV prevalence reflects MDG 6.1, and modelled using a linear probability model. Data are from UN Millennium Development Goals Indicators 2008 edition.

*p<0.05.

**p<0.01.

***p<0.001.

**Table 5:** Associations of GDP per capita, health spending/GDP, health spending, and physicians per capita with percentage of unmet progress toward health MDGs.

Covariate	Quantity of Unmet MDG Progress			
	Infant Mortality Rates	Child Mortality Rates	Tuberculosis Mortality Rates	HIV Prevalence
10% higher GDP per capita	−0.60% [−1.93 to 0.73]	−0.46% [−1.88 to 0.96]	−2.32%*** [−3.17 to −1.48]	0.78% [−0.39 to 1.94]
1% higher Health Spending as percentage of GDP	1.92% [−3.52 to 7.37]	2.19% [−3.72 to 8.11]	−1.84% [−5.48 to 1.80]	−3.31% [−7.43 to 0.82]
$10 higher Health Spending per capita (PPP)	−0.039% [−0.16 to 0.082]	−0.039% [−0.17 to 0.090]	−0.0037% [−0.095 to 0.088]	−0.084% [−0.21 to 0.038]
1 additional physician/10,000 pop.	−1.43%** [−2.49 to −0.38]	−1.44%* [−2.57 to −0.31]	0.98%** [0.24 to 1.73]	0.0093% [−0.85 to 0.86]
Number of Countries	163	163	163	131
*R* ^2^	0.190	0.152	0.215	0.045

*Notes:* Results presented from four separate regression models. Constant estimated but not reported. 95% confidence intervals based on heteroskedasticity robust standard errors in parentheses. Unmet MDG Progress is calculated in percentage terms as 100×[1–(Actual ΔMR/Expected ΔMR)]. Multiplying each coefficient by 40% transforms the coefficient to describe the associations with the percentage change in each outcome. Progress towards reducing infant mortality rates and child mortality rates reflects MDG 4.1 and 4.2 and modelled using a linear standard regression model. Progress towards halting or reversing tuberculosis mortality rates reflects MDG 6.9, and modelled using a linear probability model. Progress towards halting or reversing HIV prevalence reflects MDG 6.1, and modelled using a linear probability model. Data are from UN Millennium Development Goals Indicators 2008 edition.

*p<0.05.

**p<0.01.

***p<0.001.

### Associations of HIV and NCDs with Child Mortality Progress (MDG 4)

We next evaluated whether coexisting epidemics, specifically high burdens of HIV/AIDS or NCDs accompanying high burdens of child disease and tuberculosis, could be contributing to the MDG inequalities observed. Coexisting epidemics can create a “health trap.” That is, failure to control one disease impedes progress on the other and (possibly) vice-versa; this could occur both because of biological risks from comorbidities, and because additional household spending or loss of income resulting from one disease can deplete resources needed to combat threats to child health (e.g., ability to pay for medical care for sick children) [53] or comorbid infectious diseases (e.g., paying for transportation to refill antituberculosis medication) [54].

First, we evaluated the relationship between tuberculosis and child health MDGs and HIV prevalence among ages 15–49 y. Table 6 shows that a high background rate of HIV prevalence explains a much higher share of the inequalities between countries in health MDG progress than the other economic and health system variables. Each 1% higher HIV prevalence rate was associated with 8.46% lower progress in infant mortality (95% CI 5.57%–11.41%) and 9.25% lower progress in under-5 mortality (95% CI, 5.97%–12.5%). After adjusting for HIV prevalence, more physicians per capita had no effect on progress towards child mortality goals.

**Table 6:** Associations of GDP per capita, health spending/GDP, health spending, physicians per capita, and HIV prevalence with percentage of unmet progress toward health MDGs.

Covariate	Quantity of Unmet MDG Progress			
	Infant Mortality Rates	Child Mortality Rates	Tuberculosis Mortality Rates	HIV Prevalence
10% higher GDP per capita	−1.16% [−2.38 to 0.055]	−1.07% [−2.35 to 0.22]	−2.99%*** [−3.77 to −2.21]	0.78% [−0.38 to 1.95]
1% higher Health Spending as percentage of GDP	−3.08% [−9.16 to 3.01]	−3.12% [−9.81 to 3.57]	−4.58%* [−8.17 to −0.99]	−3.23% [−7.54 to 1.08]
$10 higher Health Spending per capita (PPP)	0.038% [−0.065 to 0.14]	0.047% [−0.079 to 0.17]	0.044% [−0.038 to 0.13]	−0.085% [−0.21 to 0.036]
1 additional physician/10,000 pop.	−0.12% [−0.91 to 0.66]	−0.031% [−0.86 to 0.80]	1.78%*** [1.02 to 2.54]	−0.0092% [−0.93 to 0.91]
1% higher HIV Prevalence	8.47%*** [5.57 to 11.4]	9.25%*** [5.97 to 12.5]	5.35%*** [4.08 to 6.62]	−0.11% [−1.87 to 1.65]
Number of Countries	131	131	131	131
*R* ^2^	0.523	0.505	0.487	0.045

*Notes:* Results presented from four separate regression models. Constant estimated but not reported. 95% confidence intervals based on heteroskedasticity robust standard errors in parentheses. Unmet MDG Progress is calculated in percentage terms as 100×[1–(Actual ΔMR/Expected ΔMR)]. Multiplying each coefficient by 40% transforms the coefficient to describe the associations with the percentage change in each outcome. Progress towards reducing infant mortality rates and child mortality rates reflects MDG 4.1 and 4.2 and modelled using a linear standard regression model. Progress towards halting or reversing tuberculosis mortality rates reflects MDG 6.9, and modelled using a linear probability model. Progress towards halting or reversing HIV prevalence reflects MDG 6.1, and modelled using a linear probability model. Data are from UN Millennium Development Goals Indicators 2008 edition.

*p<0.05.

**p<0.01.

***p<0.001

In Table 7 we included the age-standardised mortality rates from NCDs. Over and above the previous correlates of health MDG progress, we found each 10% greater NCD burden, which corresponded roughly to one standard deviation in the global country sample and has been proposed as a feasible target by WHO [55], was associated with a 6.32% reduction in progress toward infant mortality targets (95% CI 2.03%–10.6%) and a 5.78% reduction in progress toward under-5 mortality targets (95% CI 1.03%–10.5%). Our full model (Equation 2, above) depicted in Table 7 explained 56% of variation in country progress in infant mortality and 53% of variation in under-5 mortality.

**Table 7:** Associations of GDP per capita, health spending/GDP, health spending, physicians per capita, HIV prevalence, and NCD mortality rates with percentage of unmet progress toward health MDGs.

Covariate	Quantity of Unmet MDG Progress			
	Infant Mortality Rates	Child Mortality Rates	Tuberculosis Mortality Rates	HIV Prevalence
10% higher GDP per capita	−0.17% [−1.55 to 1.21]	−0.16% [−1.67 to 1.35]	−1.80%*** [−2.78 to −0.82]	1.10% [−0.29 to 2.49]
1% higher Health Spending as percentage of GDP	−2.23% [−8.71 to 4.25]	−2.35% [−9.48 to 4.78]	−3.57%* [−6.94 to −0.19]	−2.96% [−7.39 to 1.48]
$10 higher Health Spending per capita (PPP)	0.10% [−0.020 to 0.23]	0.11% [−0.050 to 0.27]	0.12%** [0.032 to 0.22]	−0.064% [−0.19 to 0.066]
1 additional physician/10,000 pop.	−0.38% [−1.25 to 0.50]	−0.26% [−1.19 to 0.66]	1.47%*** [0.75 to 2.19]	−0.091% [−1.02 to 0.84]
1% higher HIV Prevalence	8.15%*** [5.27 to 11.0]	8.95%*** [5.66 to 12.2]	4.96%*** [3.86 to 6.06]	−0.21% [−1.96 to 1.53]
10% higher NCD Mortality Rates	6.32%** [2.03 to 10.6]	5.78%* [1.03 to 10.5]	7.56%*** [4.73 to 10.4]	2.03% [−2.39 to 6.45]
Number of Countries	131	131	131	131
*R* ^2^	0.559	0.532	0.571	0.052

*Notes*: Results presented from four separate regression models. Constant estimated but not reported. 95% confidence intervals based on heteroskedasticity robust standard errors in parentheses. Unmet MDG Progress is calculated in percentage terms as 100 [1−(Actual ΔMR/Expected ΔMR)]. Multiplying each coefficient by 40% transforms the coefficient to describe the associations with the percentage change in each outcome. Progress towards reducing infant mortality rates and child mortality rates reflects MDG 4.1 and 4.2 and modelled using a linear standard regression model. Progress towards halting or reversing tuberculosis mortality rates reflects MDG 6.9, and modelled using a linear probability model. Progress towards halting or reversing HIV prevalence reflects MDG 6.1, and modelled using a linear probability model. Data are from UN Millennium Development Goals Indicators 2008 edition.

*p<0.05.

**p<0.01.

***p<0.001.

To put these associations into perspective, we found that the association between a 1% lower HIV prevalence or 10% lower NCD mortality and progress towards child mortality MDGs was of magnitude similar to a 40% rise in GDP (corresponding to at least 5 years of economic growth in low-income countries) (see Text S9 for further decomposition analysis).

### Associations of HIV and NCDs with Infectious Disease Progress (MDG #6)

Tables 6 and 7 also present the associations of HIV and NCD burdens with progress on tuberculosis and HIV MDGs. Each 1% higher HIV prevalence was associated with a 5.35% lower rate of progress on tuberculosis mortality (95% CI −6.62% to −4.08%). Each 10% higher NCD mortality was associated with a 7.56% reduction in progress toward tuberculosis mortality targets (95% CI −4.73% to 10.4%). These estimates were similar in magnitude to estimates of the population-attributable tuberculosis risk from tobacco and diabetes by Dye and colleagues at WHO [7]. We found no effect of NCDs on HIV prevalence, which would be expected because the chronic NCDs increase risks of death for persons living with HIV and, as a result, would exert a downward effect on prevalence. Including chronic NCDs also improved the fit of the models, such that our full model explained 57% of variation in tuberculosis mortality among countries. Consistent with the expectation that NCDs would not further explain HIV inequalities, the model (including rates of NCDs, baseline HIV prevalence, measures of economic development, health prioritisation, health spending, and health infrastructure) was only able to account for 5% of variations in HIV prevalence.

The association of a 1% lower HIV prevalence or 10% lower NCD burden with progress towards the tuberculosis MDG was of a similar magnitude as a 80% rise in GDP.

### Robustness Checks

We performed a series of robustness checks on both our data and model specification, as set out in Text S2–S9. This included measures of the initial disease burden, urbanization rates, and hospital beds per capita, as well as using different transformations of the predictor variables and differing samples (low HIV-prevalence according to the WHO StopTB cutpoints and low MDG progress countries), finding that the results were consistent among the various models (Text S3). We also compared the association among low-, middle-, and high-income countries; the associations between NCDs and MDG progress were consistent at all levels of income, but greatest in low-income countries where the burden of NCDs is largest (Text S4). We had some inevitable concerns about the quality of the data on adult mortality from NCDs. Although the Global Burden of Disease study (http://www.who.int/topics/global_burden_of_disease/en/), from which these data were obtained, was an enormous advance over previously available data sources, many of the data from poor countries are based on estimates derived from models that, in some cases, include child mortality derived from surveys. It is possible that this could introduce an element of circularity. We examined this possibility first by including a control for the different ways of estimating data on NCD mortality [56],[57] as well as by excluding the 42 countries in our sample that derived adult mortality from child mortality. Neither approach changed our results qualitatively or statistically (tests for effect homogeneity: χ^2^(1) = 3.60, *p* = 0.0551 and χ^2^(1) = 1.82, *p* = 0.1767 respectively) (Text S7). We also performed a series of diagnostic tests on our residuals, finding no evidence of leverage points (Cook's distance <4 in all cases) or influence points (based on leverage versus normalized squared residual plots). After removing potential outliers based on residuals greater than two standard deviations, none of our results changed (Text S5). Thus, our results were not an artefact of a few extremely poorly performing outlier countries. We also evaluated potential multicollinearity using variance inflation factors, finding that our results were within accepted statistical limits (Text S5).

## Discussion

Our results indicate that progress in achieving the health MDGs for infant and child mortality and tuberculosis is associated significantly with the burden of adult NCDs and HIV prevalence among adults aged 15–49. The evidence emerging from these models is consistent with a growing body of research finding that high burdens of NCDs contribute to worse child health and poorer tuberculosis outcomes, both as a result of biological risks from comorbidities and as a consequence of reduced household resources (in both human and financial terms) when faced with multiple comorbidities (Table 1). An abundance of evidence indicates that poor households face the greatest burdens of both HIV and NCDs (though the diagnostic infrastructure for the latter is focused on higher income groups), and are also the households most affected by child mortality and tuberculosis.

We estimated that a 1% lower HIV prevalence or 10% lower NCD risks would have an association with progress to child health MDGs similar to a 40% or greater rise in GDP, while it would equate to an 80% or greater rise in GDP for tuberculosis (corresponding to about a decade of economic growth in low-income countries). We found weaker evidence that health spending or health infrastructure, as measured by physicians per capita, was a major correlate of inequalities in progress by countries towards these MDGs. While our models explained over half of the observed inequalities among countries in progress towards child health and tuberculosis goals, a large residual in our measure of HIV/AIDS progress remained to be accounted for even after evaluating economic development, health priority, health spending, and health infrastructure as potential explanations of country inequalities.

Although researchers have sought to identify barriers to achieving progress towards MDGs in individual countries or regions (especially sub-Saharan Africa) [42],[43], this study is, to our knowledge, the first attempt to analyse and compare the determinants of global inequalities in progress to the health MDGs. Inevitably, this initial analysis has several important limitations which are relevant both to future research and to the updates to the MDG process scheduled for 2010.

First, a comparative analysis of progress towards achievement of health MDGs risks generating ecologic fallacies where the unit of analysis is the country. However, our findings are consistent with a large body of micro-level evidence indicating the importance of NCDs among adults for the health of their dependent children and for the control of infectious diseases (Table 1), as well as the role of HIV in both child health and infectious disease MDGs.

Second, we cannot be certain that the associations we have observed between HIV prevalence, NCDs, and progress towards health MDGs are causal. One limitation is that we use cross-sectional data to infer longitudinal relationships, making it possible to observe a correlation cross-sectionally across countries which does not occur longitudinally within individual country time series. However, we have taken advantage of longitudinal data to evaluate the rate of progress, or change, in MDG indicators. We also need to consider two other possibilities: that either (i) poor performance on health MDGs contributes to growing levels of NCDs; or (ii) a third underlying factor is implicated in both poor chronic disease outcomes and slow progress on health MDGs. Beginning with the first mechanism, although there is now extensive evidence from the field of life course epidemiology linking adverse conditions in childhood to several NCDs in adulthood, the effects are, by their nature, seen only after a lag of several decades. Also, while some NCDs have established infectious aetiologies, such as cervical cancer and peptic ulcers, and inflammation related to infections may play a role in ischaemic heart disease [58],[59] and diabetes [60], these infectious agents make a relatively small contribution to the total burden of NCDs, compared to the major risk factors of poor diet, low physical activity, smoking, and alcohol. Hence, the first mechanism seems implausible. It is impossible to exclude the second in a cross-sectional analysis but we tested the most plausible such factors (poverty, health system financing, and health care infrastructure) and found a significant relationship between HIV/AIDS, chronic diseases, and health MDGs independently of these.

Third, country-wide indicators potentially mask within-country inequalities [61]. In the future it will be important for the UN to obtain data that can be disaggregated into different groups within populations, as the determinants of inequalities in MDG progress within-country may differ from the drivers of between-country inequalities that our study has investigated.

Finally, the data available to assess MDG progress from existing sources are limited. Although the UN database is the most comprehensive source, many important health system performance indicators are missing on a comparative longitudinal basis. Time series data relating to many health MDGs in low-income countries are presently incomplete, in particular maternal mortality ratios and HIV prevalence. This limited the number of health indicators we were able to study. Of the data which do exist from poor countries, such as infant and under-5 mortality rates, most are based on demographic projections from sentinel sites or small surveys. Scaling up surveillance will be critical for a better understanding of each country's performance. If the quality of these data is subject to error, we would expect regression to the mean to reduce the likelihood of finding such robust and strongly significant findings through so many tests and alternative model specifications. Yet the findings remained robust. Also, in spite of the important limitations of the existing data, we note that the indicators evaluated in this analysis are the same data followed by the UN. The ability to conduct an exercise such as this one—to evaluate why some countries have been more successful than others on the same targets—is a key benefit of the MDG process. Similarly, caution is needed in using the data on NCDs from the Global Burden of Disease study, although as we showed, our findings are robust to exclusion of those countries where there may be a degree of circularity because the estimates incorporate data on childhood mortality.

These findings have implications for global health policy. The dichotomy between adult noncommunicable mortality and the measures included in the MDGs (communicable diseases and child mortality) may obscure the interrelationships of illnesses affecting those living in poor households. Programs designed to achieve the existing health MDGs should take account of the relationships among all those diseases that can trap households in vicious cycles of mortality and poverty. Global health initiatives are now placing a much greater emphasis on health systems strengthening. However, in many cases, these are seen as a means to achieving their specific goals, such as improving uptake of immunization [62] or delivering antiretrovirals [63]. Our analysis, indicating correlations among diseases and their risks, suggests that their activities should be broadened to address the burden of other disorders that may also, albeit less directly, impact on their ability to achieve their goals.

Our findings also have implications for future research. There is a need to investigate why and how the burden of HIV and chronic NCDs may affect progress on child health and tuberculosis MDGs. Further expanding data availability is crucially needed to allow for more robust time series and panel data analysis in the future, so that longitudinal relationships among joint epidemics can be tracked with greater confidence.

Our findings suggest that achievement of feasible reductions in the impact of these chronic diseases on poor households could greatly enhance progress towards existing health MDGs. If not adequately addressed, high rates of NCDs in low-income countries may further impede progress towards the health MDGs.

## Supporting Information

Text S1 Years of MDG data availability.
(0.08 MB DOC)

Text S2 Representative unadjusted values, GDP.
(0.06 MB DOC)

Text S3 Initial conditions and health MDG progress.
(0.14 MB DOC)

Text S4 Unmet progress towards MDG #4, child health and chronic non-communicable disease mortality rates, by income.
(0.03 MB DOC)

Text S5 Model diagnostics.
(0.12 MB DOC)

Text S6 More details on the relationship between chronic NCDs and health MDGs.
(0.05 MB DOC)

Text S7 Surveillance robustness check.
(0.06 MB DOC)

Text S8 Sample alternative box plots.
(0.03 MB DOC)

Text S9 Sample predicted progress based on NCD and HIV rates.
(0.03 MB DOC)

## Abbreviations

GDP: gross domestic product
MDG: Millennium Development Goal
NCD: noncommunicable disease
