# Supplementary material for: Drivers of Inequality in Millennium Development Goal Progress: A Statistical Analysis
Source: PLoS Med. 2010 Mar 2;7(3):e1000241. doi: 10.1371/journal.pmed.1000241 (PMC2830449; doi:10.1371/journal.pmed.1000241)
Supplement: Text S1 — Years of MDG data availability. (0.08 MB DOC) [file pmed.1000241.s001.doc]

Text S1. Years of MDG Data Availability

| Health MDG Target | Description | Country-Years of Data Availability | Mean | Std. Dev | Min | Max |
| --- | --- | --- | --- | --- | --- | --- |
| ***4.1*** | ***Children under five mortality rate per 1,000 live births*** | ***965*** | ***63.1*** | ***66.2*** | ***3.0*** | ***320.0*** |
| ***4.2*** | ***Infant mortality rate (0-1year) per 1,000 live births*** | ***965*** | ***43.6*** | ***40.1*** | ***2.0*** | ***191.0*** |
| 4.3 | Children 1 year old immunized against measles, percentage | 3171 | 80.8 | 18.2 | 8.0 | 99.0 |
| 5.1 | Maternal mortality ratio per 100,000 live births | 169 | 313.7 | 413.7 | 1.0 | 2100.0 |
| 5.2 | Births attended by skilled health personnel, percentage | 689 | 80.0 | 26.7 | 5.0 | 100.0 |
| 5.3 | Current contraceptive use among married women 15-49 years old, any method, percentage | 473 | 46.6 | 23.4 | 1.7 | 89.0 |
| 5.4 | Adolescent birth rate, per 1,000 women | 1983 | 46.7 | 37.3 | 0.0 | 228.0 |
| 5.5 | Antenatal care coverage, at least one visit, percentage | 342 | 77.8 | 20.8 | 15.4 | 100.0 |
| 5.5 | Antenatal care coverage, at least four visits, percentage | 68 | 55.4 | 23.6 | 11.4 | 96.8 |
| 5.6 | Unmet need for family planning, total, percentage | 211 | 19.6 | 9.6 | 1.3 | 47.8 |
| ***6.1*** | ***People living with HIV, 15-49 years old, percentage*** | ***286*** | ***2.6*** | ***7.2*** | ***0.1*** | ***75.1*** |
| 6.2 | Condom use at last high-risk sex, 15-24 years old, men, percentage | 72 | 48.9 | 15.8 | 12.0 | 88.0 |
| 6.2 | Condom use at last high-risk sex, 15-24 years old, women, percentage | 91 | 34.8 | 16.9 | 5.0 | 75.0 |
| 6.3 | Men 15-24 years old with comprehensive correct knowledge of HIV/AIDS, percentage | 55 | 29.1 | 13.3 | 0.0 | 54.0 |
| 6.3 | Women 15-24 years old with comprehensive correct knowledge of HIV/AIDS, percentage | 93 | 25.2 | 14.5 | 1.0 | 60.0 |
| 6.4 | Ratio of school attendance rate of orphans to school attendance rate of non orph | 121 | 0.9 | 0.1 | 0.2 | 1.1 |
| 6.5 | Antiretroviral therapy coverage among people with advanced HIV infection, percentage | 211 | 31.0 | 22.3 | 0.0 | 95.0 |
| 6.6 | Malaria death rate per 100,000 population, ages 0-4 | 0 | 0.0 | 0.0 | 0.0 | 0.0 |
| 6.7 | Children under 5 sleeping under insecticide-treated bed nets, percentage | 74 | 8.7 | 11.4 | 0.1 | 50.3 |
| 6.8 | Children under 5 with fever being treated with anti-malarial drugs, percentage | 80 | 35.3 | 22.4 | 0.2 | 68.8 |
| ***6.9*** | ***Tuberculosis death rate per year per 100,000 population*** | ***2693*** | ***26.3*** | ***37.1*** | ***0.2*** | ***356.7*** |
| ***6.9*** | ***Tuberculosis prevalence rate per year per 100,000 population*** | ***2693*** | ***200.1*** | ***236.9*** | ***1.9*** | ***1489.9*** |
| ***6.9*** | ***Tuberculosis incidence rate per year per 100,000 population*** | ***2693*** | ***128.6*** | ***154.7*** | ***2.3*** | ***1155.3*** |
| 6.10 | Tuberculosis treatment success rate under DOTS, percentage | 1545 | 76.7 | 14.9 | 0.0 | 100.0 |
| None | Children under 5 moderately or severely underweight (%) | 425 | 19.0 | 13.8 | 0.6 | 67.4 |
| None | Population undernourished, percentage | 408 | 19.5 | 16.4 | 2.5 | 73.0 |
