# Supplementary material for: Drivers of Inequality in Millennium Development Goal Progress: A Statistical Analysis
Source: PLoS Med. 2010 Mar 2;7(3):e1000241. doi: 10.1371/journal.pmed.1000241 (PMC2830449; doi:10.1371/journal.pmed.1000241)
Supplement: Text S2 — Representative unadjusted values, GDP. (0.06 MB DOC) [file pmed.1000241.s002.doc]

**Text S2. Representative Unadjusted Figures, GDP**

Figure A. Unmet Progress towards MDG #4 Child Health and Economic Development

*Notes:* Data are from the UN Millennium Development Goals Indicators, available at <http://mdgs.un.org/unsd/mdg/Default.aspx>. Unmet MDG Progress is calculated in percentage terms as 100 * [1 – (Actual ∆MR/Expected ∆MR)]. MDG #4 aims to reduce infant and child mortality by two-thirds by 2015. Unmet progress>100 denotes worsening outcomes (i.e., reverse progress). Note that a one unit increase in log GDP per capita corresponds to more than a doubling of GDP per capita.

Figure B. Model-Based Estimates of the Probability of Falling Behind on MDG #6 Infectious Diseases and Economic Development

*Notes:* Data are from the UN Millennium Development Goals Indicators, available at <http://mdgs.un.org/unsd/mdg/Default.aspx>. Unmet MDG Progress is based on a model of the probability of achieving MDG #6, “to halt and reverse the incidence, prevalence and mortality of HIV, malaria and other diseases [including tuberculosis]”, specified as Pr(Unmet MDG #6 Progress*_i_*) = Φ(α +β_1_NCD*_i_*). Countries which experienced rises in tuberculosis mortality rates or HIV prevalence rates were assigned a one, denoting unmet progress, whereas those which had no change or reduction were assigned a zero. Note that a one unit increase in log GDP per capita corresponds to more than a doubling of GDP per capita.

Figure C. Unmet MDG #4 Progress and Chronic NCDs

*Notes:* Data are from the UN Millennium Development Goals Indicators, available at <http://mdgs.un.org/unsd/mdg/Default.aspx>. Unmet MDG Progress is calculated in percentage terms as 100 * [1 – (Actual ∆MR/Expected ∆MR)]. MDG #4 aims to reduce infant and child mortality by two-thirds by 2015. Unmet progress>100% denotes worsening outcomes (i.e., reverse progress).

Figure D. Model-Based Estimates of the Probability of Achieving MDG #6 and Chronic NCDs

*Notes:* Data are from the UN Millennium Development Goals Indicators, available at <http://mdgs.un.org/unsd/mdg/Default.aspx>. Unmet MDG Progress is based on a model of the probability of achieving MDG #6, “to halt and reverse the incidence, prevalence and mortality of HIV, malaria and other diseases [including tuberculosis]”, specified as Pr(Unmet MDG #6 Progress*_i_*) = Φ(α +β_1_NCD*_i_*). Countries which experienced rises in tuberculosis mortality rates or HIV prevalence rates were assigned a one, denoting unmet progress, whereas those which had no change or reduction were assigned a zero.

Low-income countries, where the burden of chronic diseases is greatest, appear to have the most to gain. For example, if death rates from chronic diseases were reduced 10% from the average rate for low-income countries, of 824 per 100,000, to 741 per 100,000, it is estimated that the probability of achieving MDG#6 would increase from 27.5% to 35.8%. In high income countries, where the chronic disease burden is 560 per 100,000, a 10% reduction would correspond to a 7.4% boost in the chances of MDG success, from 56.2% to 62.3%.
