# Supplementary material for: Drivers of Inequality in Millennium Development Goal Progress: A Statistical Analysis
Source: PLoS Med. 2010 Mar 2;7(3):e1000241. doi: 10.1371/journal.pmed.1000241 (PMC2830449; doi:10.1371/journal.pmed.1000241)
Supplement: Text S3 — Initial conditions and health MDG progress. (0.14 MB DOC) [file pmed.1000241.s003.doc]

**Text S3. Initial Conditions and Health MDG Progress**

Another possibility is that countries with worse health at the time the MDGs were formulated will simply achieve less MDG progress. For example, it may be the case that those countries resistant to progress in child mortality also have high levels of child mortality. We can correct for this scenario by incorporating the initial rates of mortality into our models.

As shown below, we found that higher initial rates of infant, under-5 and tuberculosis mortality rates and HIV prevalence were significantly associated with slower progress toward the MDG goals (Table A). For example, each 10 higher infant deaths per 1,000 live births a country at baseline was linked on average to 4.1% greater unmet MDG progress in the future. Yet, after considering worse initial health conditions (table below) and economic development (Table 2 main text) as possible explanations for slow progress in low-income countries, over three-quarters of the cross-country inequalities remained unaccounted for. That is, a vast majority of the factors explaining why two countries would experience different progress on the public health MDGs could not be accounted for by economic development or initial health conditions.

Table A. Associations of initial health conditions with percentage of unmet progress toward Health-Related Millennium Development Goals

|  | Quantity of Unmet MDG Progress | | | |
| --- | --- | --- | --- | --- |
|  | Infant Mortality Rates | Child Mortality Rates | Tuberculosis Mortality Rates | HIV Prevalence |
| Initial Rates^†^ | 0.41^***^ [0.29,0.52] | 0.25^***^ [0.18,0.32] | 0.34^**^ [0.096,0.58] | -0.72 [-1.90,0.46] |
| Number of Countries | 191 | 191 | 206 | 142 |
| *R*^2^ | 0.135 | 0.128 | 0.044 | 0.006 |

*Notes:* Results presented from four separate regression models. Constant estimated but not reported. 95% confidence intervals in parentheses based on robust standard errors. Unmet MDG Progress is calculated in percentage terms as 100 * [1 – (Actual ∆MR/Expected ∆MR)]. Progress towards reducing infant mortality rates and child mortality rates reflects MDG Targets 4.1 and 4.2. Progress towards halting or reversing tuberculosis mortality rates reflects MDG Target 6.9. Progress towards halting or reversing HIV prevalence reflects MDG Target 6.1. ^†^ - Infant mortality is deaths per 1,000 live births. Child mortality is deaths per 1,000 children under age-5. Tuberculosis mortality is scaled to per 100,000 population. HIV Prevalence is the percentage of the population living with HIV/AIDS between ages 15 to 49. Data are from Millennium Development Goals Indicators 2008 edition.

^*^ *p* < 0.05, ^**^ *p* < 0.01, ^***^ *p* < 0.001

*Additional Models*

Table B. Unadjusted associations of a 10% reduction in age-standardised chronic non-communicable diseases (NCDs) with percentage of unmet progress toward Health MDGs

|  | Quantity of Unmet MDG Progress | | | |
| --- | --- | --- | --- | --- |
|  | Infant Mortality | Child Mortality | TB Mortality | HIV Prevalence |
| 10% higher Chronic NCDs | 5.37%^***^ [3.95,6.79] | 5.08%^***^ [3.59,6.57] | 7.11^***^ [5.39,8.82] | 0.95 [-1.47,3.37] |
| Number of Countries | 188 | 188 | 189 | 141 |
| *R*^2^ | 0.123 | 0.101 | 0.224 | 0.004 |

*Notes:* Results presented from four separate regression models. Constant estimated but not reported. 95% confidence intervals in parentheses based on robust standard errors. Unmet MDG Progress is calculated in percentage terms as 100 * [1 – (Actual ∆MR/Expected ∆MR)]. Progress towards reducing infant mortality rates and child mortality rates reflects MDG Targets 4.1 and 4.2. Progress towards halting or reversing tuberculosis mortality rates reflects MDG Target 6.9. Progress towards halting or reversing HIV prevalence reflects MDG Target 6.1. Data are from Millennium Development Goals Indicators 2008 edition.

^*^ *p* < 0.05, ^**^ *p* < 0.01, ^***^ *p* < 0.001

Table C. Adjusted associations of a 10% reduction in age-standardised chronic non-communicable diseases (NCDs) with percentage of unmet progress toward Health MDGs

|  | Quantity of Unmet MDG Progress | | | |
| --- | --- | --- | --- | --- |
|  | Infant Mortality | Child Mortality | TB Mortality | HIV Prevalence |
| 10% higher Chronic NCDs | 3.64%^*^ [0.11,7.16] | 3.51% [-0.045,7.06] | 6.96%^***^ [3.98,9.94] | 3.05% [-1.13,7.23] |
| 10% higher GDP per capita | -0.58% [-1.55,0.39] | -0.53% [-1.51,0.46] | -0.28% [-1.14,0.57] | 0.65% [-0.45,1.75] |
| Number of Countries | 164 | 164 | 165 | 138 |
| *R*^2^ | 0.123 | 0.101 | 0.255 | 0.018 |

*Notes:* Results presented from four separate regression models. Constant estimated but not reported. 95% confidence intervals in parentheses based on robust standard errors. Unmet MDG Progress is calculated in percentage terms as 100 * [1 – (Actual ∆MR/Expected ∆MR)]. Progress towards reducing infant mortality rates and child mortality rates reflects MDG Targets 4.1 and 4.2. Progress towards halting or reversing tuberculosis mortality rates reflects MDG Target 6.9. Progress towards halting or reversing HIV prevalence reflects MDG Target 6.1. Data are from Millennium Development Goals Indicators 2008 edition.

^*^ *p* < 0.05, ^**^ *p* < 0.01, ^***^ *p* < 0.001

Table D. Adjusted associations of a 10% reduction in age-standardised chronic non-communicable diseases (NCDs) with percentage of unmet progress toward Health MDGs

|  | Quantity of Unmet MDG Progress | | | |
| --- | --- | --- | --- | --- |
|  | Infant Mortality | Child Mortality | TB Mortality | HIV Prevalence |
| 10% higher Chronic NCDs | 2.89% [-0.39,6.17] | 2.80% [-0.46,6.05] | 6.96%^***^ [3.97,9.94] | 3.13% [-1.09,7.36] |
| 10% higher GDP per capita | 0.18% [-1.27,1.62] | 0.41% [-1.01,1.83] | -0.23% [-1.21,0.74] | 0.60% [-0.51,1.71] |
| Initial Rates | 0.29% [-0.041,0.62] | 0.21%^*^ [0.029,0.40] | 0.034% [-0.33,0.40] | -0.80% [-2.00,0.41] |
| Number of Countries | 164 | 164 | 165 | 132 |
| *R*^2^ | 0.141 | 0.128 | 0.256 | 0.025 |

*Notes:* Results presented from four separate regression models. Constant estimated but not reported. 95% confidence intervals in parentheses based on robust standard errors. Unmet MDG Progress is calculated in percentage terms as 100 * [1 – (Actual ∆MR/Expected ∆MR)]. Progress towards reducing infant mortality rates and child mortality rates reflects MDG Targets 4.1 and 4.2. Progress towards halting or reversing tuberculosis mortality rates reflects MDG Target 6.9. Progress towards halting or reversing HIV prevalence reflects MDG Target 6.1. Data are from Millennium Development Goals Indicators 2008 edition.

^*^ *p* < 0.05, ^**^ *p* < 0.01, ^***^ *p* < 0.001

Table E. Adjusted associations of a 10% reduction in age-standardised chronic non-communicable diseases (NCDs) with percentage of unmet progress toward Health-Related MDGs

|  | Quantity of Unmet MDG Progress | | | |
| --- | --- | --- | --- | --- |
|  | Infant Mortality | Child Mortality | TB Mortality | HIV Prevalence |
| 10% higher Chronic NCDs | 2.49% [-0.88,5.86] | 2.17% [-1.13,5.46] | 8.23%^***^ [4.99,11.5] | 2.19% [-2.24,6.61] |
| 10% higher GDP per capita | 0.53% [-1.36,2.42] | 0.92% [-0.97,2.82] | -0.69% [-1.78,0.39] | 0.90% [-0.52,2.33] |
| Initial Rates | 0.30% [-0.050,0.65] | 0.23%^*^ [0.028,0.43] | 0.11% [-0.25,0.47] | -0.86% [-2.11,0.38] |
| Urbanisation Rates | -0.19% [-0.66,0.28] | -0.24% [-0.73,0.25] | 0.21% [-0.17,0.59] | 0.043% [-0.50,0.58] |
| Health Spending per capita (PPP) | -0.028% [-0.100,0.044] | -0.046% [-0.12,0.032] | 0.085%^*^ [0.011,0.16] | -0.11% [-0.23,0.013] |
| Number of Countries | 163 | 163 | 163 | 131 |
| *R*^2^ | 0.148 | 0.138 | 0.289 | 0.047 |

*Notes:* Results presented from four separate regression models. Constant estimated but not reported. 95% confidence intervals in parentheses based on robust standard errors. Unmet MDG Progress is calculated in percentage terms as 100 * [1 – (Actual ∆MR/Expected ∆MR)]. Progress towards reducing infant mortality rates and child mortality rates reflects MDG Targets 4.1 and 4.2. Progress towards halting or reversing tuberculosis mortality rates reflects MDG Target 6.9. Progress towards halting or reversing HIV prevalence reflects MDG Target 6.1. Data are from Millennium Development Goals Indicators 2008 edition.

^*^ *p* < 0.05, ^**^ *p* < 0.01, ^***^ *p* < 0.001

Table F. Log Transformation of Health Spending

|  | (1) | (2) | (3) | (4) |
| --- | --- | --- | --- | --- |
|  | Unmet Infant Mortality Progress | Unmet Child Mortality Progress | Unmet TB Progress | Unmet HIV Progress |
| Log GDP per capita | -1.34 [-3.06,0.39] | -1.18 [-3.03,0.67] | -1.16 [-2.52,0.19] | 1.36 [-0.43,3.16] |
| Health/GDP | 0.19 [-4.73,5.11] | 0.48 [-4.80,5.76] | 0.54 [-3.32,4.41] | -2.65 [-7.90,2.60] |
| Log Health Spending per capita | -3.99 [-18.2,10.3] | -4.13 [-19.4,11.2] | -4.59 [-15.8,6.62] | -8.40 [-23.0,6.21] |
| Observations | 163 | 163 | 163 | 131 |
| *R*^2^ | 0.132 | 0.100 | 0.177 | 0.044 |

*Notes:* Untransformed coefficients presented. 95% confidence intervals in brackets

^*^ *p* < 0.05, ^**^ *p* < 0.01, ^***^ *p* < 0.001

Table G. Associations of GDP per capita and Health spending/GDP with percentage of unmet progress toward Health-Related Millennium Development Goals

|  | Quantity of Unmet MDG Progress | | | |
| --- | --- | --- | --- | --- |
|  | Infant Mortality Rates | Child Mortality Rates | Tuberculosis Mortality Rates | HIV Prevalence |
| 10% higher GDP per capita | 0.40 [-1.22,2.02] | 0.47 [-1.26,2.21] | -1.15^*^ [-2.18,-0.12] | 1.08 [-0.31,2.47] |
| 1% higher Health Spending as percentage of GDP | 2.47 [-3.25,8.19] | 2.70 [-3.53,8.93] | -1.19 [-4.70,2.33] | -3.11 [-7.31,1.08] |
| $10 higher Health Spending per capita (PPP) | 0.023 [-0.097,0.14] | 0.019 [-0.12,0.15] | 0.072 [-0.017,0.16] | -0.062 [-0.19,0.067] |
| 1 additional physician/10,000 pop. | -1.64^**^ [-2.73,-0.55] | -1.63^**^ [-2.80,-0.46] | 0.76^*^ [0.10,1.41] | -0.053 [-0.92,0.81] |
| 10% higher NCD Mortality Rates | 6.16^**^ [1.77,10.6] | 5.74^*^ [1.08,10.4] | 7.41^***^ [4.34,10.5] | 1.97 [-2.41,6.36] |
| Number of Countries | 163 | 163 | 163 | 131 |
| *R*^2^ | 0.231 | 0.184 | 0.306 | 0.051 |

*Notes:* Results presented from four separate regression models. Constant estimated but not reported. 95% confidence intervals in parentheses. Unmet MDG Progress is calculated in percentage terms as 100 * [1 – (Actual ∆MR/Expected ∆MR)]. Progress towards reducing infant mortality rates and child mortality rates reflects MDG Targets 4.1 and 4.2 and modeled using a linear standard regression model. Progress towards halting or reversing tuberculosis mortality rates reflects MDG Target 6.9, and modeled using a linear probability model. Progress towards halting or reversing HIV prevalence reflects MDG Target 6.1, and modeled using a linear probability model. Data are from UN Millennium Development Goals Indicators 2008 edition.

^*^ *p* < 0.05, ^**^ *p* < 0.01, ^***^ *p* < 0.001

Table H: Full Model, countries <4% HIV prevalence, untransformed coefficients.

|  | (1) | (2) | (3) | (4) |
| --- | --- | --- | --- | --- |
|  | Unmet Infant Mortality Progress | Unmet Child Mortality Progress | Unmet TB Progress | Unmet HIV Progress |
| 10% higher GDP per capita | -0.72 [-2.14,0.70] | -0.66 [-2.20,0.88] | -1.85^***^ [-2.88,-0.83] | 0.76 [-0.79,2.30] |
| 1% higher Health Spending as percentage of GDP | -1.27 [-6.58,4.04] | -1.23 [-7.01,4.55] | -3.79^*^ [-7.03,-0.56] | -2.58 [-7.76,2.59] |
| $10 higher Health Spending per capita (PPP) | 0.074 [-0.024,0.17] | 0.070 [-0.056,0.20] | 0.11^*^ [0.012,0.20] | -0.060 [-0.19,0.073] |
| 1 additional physician/10,000 pop. | -0.0011 [-0.85,0.85] | 0.14 [-0.77,1.04] | 1.81^***^ [1.06,2.56] | 0.025 [-0.95,1.00] |
| 10% higher NCD Mortality Rates | 3.83 [-0.45,8.10] | 3.17 [-1.44,7.78] | 6.13^***^ [3.36,8.91] | 1.35 [-3.36,6.05] |
| 1% higher HIV Prevalence (People living with HIV, 15-49 years old, percentage) | 22.0^***^ [12.5,31.6] | 24.4^***^ [13.6,35.3] | 16.4^***^ [8.06,24.8] | 3.00 [-7.90,13.9] |
| Number of Countries | 116 | 116 | 116 | 116 |
| *R*^2^ | 0.440 | 0.389 | 0.547 | 0.042 |

95% confidence intervals in brackets

^*^ *p* < 0.05, ^**^ *p* < 0.01, ^***^ *p* < 0.001

Table I: Unadjusted Model including hospital beds/capita, untransformed coefficients.

|  | (1) | (2) | (3) | (4) |
| --- | --- | --- | --- | --- |
|  | Unmet Infant Mortality Progress | Unmet Child Mortality Progress | Unmet TB Progress | Unmet HIV Progress |
| 1 additional Hospital Bed per capita | -0.68^***^ [-0.95,-0.41] | -0.60^***^ [-0.89,-0.31] | -0.11 [-0.40,0.19] | -0.036 [-0.32,0.24] |
| Constant | 42.6^***^ [29.4,55.9] | 36.0^***^ [21.5,50.6] | 36.1^***^ [24.2,48.0] | 30.4^***^ [18.6,42.3] |
| Observations | 175 | 175 | 177 | 133 |
| *R*^2^ | 0.088 | 0.062 | 0.003 | <0.001 |

95% confidence intervals in brackets

^*^ *p* < 0.05, ^**^ *p* < 0.01, ^***^ *p* < 0.001

Table J: Adjusted Model including hospital beds/capita, untransformed coefficients.

|  | (1) | (2) | (3) | (4) |
| --- | --- | --- | --- | --- |
|  | Unmet Infant Mortality Progress | Unmet Child Mortality Progress | Unmet TB Progress | Unmet HIV Progress |
| 10% higher GDP per capita | 0.14 [-1.31,1.58] | 0.12 [-1.47,1.71] | -1.90^**^ [-3.01,-0.78] | 1.40 [-0.030,2.83] |
| 1% higher Health Spending as percentage of GDP | -3.36 [-9.67,2.95] | -3.51 [-10.4,3.42] | -3.62^*^ [-7.05,-0.18] | -2.89 [-7.28,1.49] |
| $10 higher Health Spending per capita (PPP) | 0.096 [-0.033,0.22] | 0.089 [-0.071,0.25] | 0.11^*^ [0.017,0.20] | -0.089 [-0.22,0.039] |
| 1 additional physician/10,000 pop. | -0.39 [-1.38,0.60] | -0.44 [-1.47,0.59] | 0.97^**^ [0.25,1.68] | 0.085 [-0.89,1.06] |
| 1% higher HIV Prevalence (People living with HIV, 15-49 years old, percentage) | 6.06^**^ [1.71,10.4] | 5.30^*^ [0.50,10.1] | 7.13^***^ [4.38,9.89] | 1.58 [-2.81,5.97] |
| 10% higher NCD Mortality Rates | 8.17^***^ [5.25,11.1] | 8.93^***^ [5.60,12.3] | 4.74^***^ [3.68,5.81] | 0.0077 [-1.79,1.80] |
| Hospital Beds per capita | -0.060 [-0.48,0.36] | 0.070 [-0.37,0.51] | 0.37^**^ [0.10,0.63] | -0.10 [-0.53,0.32] |
| Observations | 124 | 124 | 124 | 124 |
| *R*^2^ | 0.568 | 0.545 | 0.578 | 0.060 |

95% confidence intervals in brackets. Constant included but not reported. Untransformed coefficients presented.

^*^ *p* < 0.05, ^**^ *p* < 0.01, ^***^ *p* < 0.001

Table K. Bad Performers (>20% Unmet MDG Progress)

|  | (1) | (2) |
| --- | --- | --- |
|  | Unmet Infant Mortality Progress | Unmet Child Mortality Progress |
| 10% higher GDP per capita | 0.93 [-0.47,2.34] | 1.02 [-0.67,2.70] |
| 1% higher Health Spending as percentage of GDP | -0.12 [-7.27,7.03] | -0.36 [-8.89,8.17] |
| $10 higher Health Spending per capita (PPP) | 0.053 [-0.30,0.40] | 0.077 [-0.35,0.50] |
| 1 additional physician/10,000 pop. | -0.33 [-1.42,0.75] | -0.39 [-1.69,0.91] |
| 1% higher HIV Prevalence (People living with HIV, 15-49 years old, percentage) | 7.24^*^ [1.29,13.2] | 6.71 [-1.02,14.4] |
| 10% higher NCD Mortality Rates | 6.42^***^ [4.66,8.17] | 6.85^***^ [4.71,9.00] |
| Observations | 55 | 51 |
| *R*^2^ | 0.625 | 0.578 |

95% confidence intervals in brackets

^*^ *p* < 0.05, ^**^ *p* < 0.01, ^***^ *p* < 0.001
