# Supplementary material for: Drivers of Inequality in Millennium Development Goal Progress: A Statistical Analysis
Source: PLoS Med. 2010 Mar 2;7(3):e1000241. doi: 10.1371/journal.pmed.1000241 (PMC2830449; doi:10.1371/journal.pmed.1000241)
Supplement: Text S4 — Unmet progress towards MDG #4, child health and chronic non-communicable disease mortality rates, by income. (0.03 MB DOC) [file pmed.1000241.s004.doc]

**Text S4. Unmet Progress towards MDG #4 Child Health and Chronic Non-Communicable Disease Mortality Rates, By Income**

Figure Associations of Chronic Noncommunicable Disease Mortality Rates with Unmet MDG Progress, by Income Group
