# Supplementary material for: Drivers of Inequality in Millennium Development Goal Progress: A Statistical Analysis
Source: PLoS Med. 2010 Mar 2;7(3):e1000241. doi: 10.1371/journal.pmed.1000241 (PMC2830449; doi:10.1371/journal.pmed.1000241)
Supplement: Text S5 — Model diagnostics. (0.12 MB DOC) [file pmed.1000241.s005.doc]

**Text S5. Model Diagnostics**

Added-Variable Plots, Progress in Infant Mortality

*Note:* Leverage is assessed as Cook’s distance, plotted on the y-axis

We did not find evidence of influence points (as evidence by no data points in upper-right hand corner of the leverage versus squared residual plot), which was consistent with observations in the added-variable plots.

Residual-versus-fitted plots

However, in examining residual-versus-fitted plots, we did see some evidence of heteroskedasticity at little or reverse progress.

This could suggest important heterogeneity, such that the predictors have greater effects on these countries, or model mis-specification, such that other political factors such as war or corruption could play a role in these countries. Examining such political determinants remains an important role for future research, especially for progress in HIV/AIDS which was virtually unexplained by the leading explanations of inequalities in health MDG progress. We found using a Szroeter’s rank test for heteroskedasticity that the heteroskedasticity appeared to arise from the HIV prevalence covariate, as shown below.

| Variable | χ^2^ | Df | p-value |
| --- | --- | --- | --- |
| GDP per capita | 0.99 | 1 | 0.3187 |
| Health/GDP | 0.33 | 1 | 0.5677 |
| Health Expenditure (PPP) | 0.31 | 1 | 0.5803 |
| Physicians per capita | 5.74 | 1 | 0.0166 |
| HIV Antenatal Prevalence | 10.62 | 1 | 0.0011 |
| Log NCD mortality rates | 1.5 | 1 | 0.2205 |

*Notes:* H_0_: constant variance; H_a_: variance monotonic

As is well-known, heteroskedasticity does not affect the coefficients but the inference. One standard approach is to adjust the standard errors for robustness in the presence of heteroskedasticity. Using a Huber/White sandwich estimator, we found all of our results were statistically unchanged and, in most cases, became slightly more significant.

Another possible issue is multicollinearity. However, as shown in the correlation matrix, none of the bivariate correlations is above 0.80, a commonly adopted threshold for concerns about multi-collinearity.

Correlation Matrix

|  | Unmet IMR progress | GDP per cap | Health/GDP | Health Exp | Physician | HIV |
| --- | --- | --- | --- | --- | --- | --- |
| Unmet IMR progress | 1 |  |  |  |  |  |
| GDP per capita | -0.37 | 1 |  |  |  |  |
| Health/GDP | -0.19 | 0.50 | 1 |  |  |  |
| Health Expenditure (PPP) | -0.31 | 0.73 | 0.63 | 1 |  |  |
| Physicians per capita | -0.42 | 0.70 | 0.55 | 0.61 | 1 |  |
| HIV Antenatal Prevalence | 0.67 | -0.17 | 0.01 | -0.18 | -0.34 | 1 |
| Log NCD mortality rates | 0.42 | -0.75 | -0.49 | -0.70 | -0.50 | 0.17 |

We also present the variance inflation factors for our full model below. VIF range from 1.0 to infinity, and scores greater than 10.0 are generally seen as indicative of severe multicollinearity. Tolerance, or 1/VIF, ranges from 0.0 to 1.0, with 1.0 being the absence of multicollinearity.

| Variable | VIF | 1/VIF (Tolerance) |
| --- | --- | --- |
| GDP per capita | 3.71 | 0.27 |
| Log NCD mortality rates | 2.96 | 0.34 |
| Health Expenditure (PPP) | 2.75 | 0.36 |
| Physicians per capita | 2.61 | 0.38 |
| Health/GDP | 1.95 | 0.51 |
| HIV Antenatal Prevalence | 1.25 | 0.80 |
| Mean VIF | 2.54 |  |

As shown in the table, none of our variables is above the commonly applied 10 threshold used to indicate multicollinearity.[1]

We tested each variable for skewness, finding the appropriate normal transformation was log for GDP and NCD data, as shown in the figure below for GDP.

Alternative Estimation Methods

An alternative, yet equivalent, statistical approach would be to model the untransformed, real values of the data, then offset the data to correspond to unmet MDG progress. In the case of GDP, we would have:

∆IMR_i_ = α + βGDP_i_ + ε_i_

Here *i* is country and ∆IMR is the percentage change in infant mortality rates. Our estimated coefficient, β, would then describe the association of GDP with the percentage change in infant mortality rates.

We obtain:

|  | (1) |
| --- | --- |
|  | Percentage Change in Infant Mortality Rates |
| 10% higher GDP per capita (PPP) | -0.72%^***^ [-1.01,-0.43] |
| Constant | 29.5%^*^ [4.41,54.6] |
| Observations | 164 |
| *R*^2^ | 0.130 |

95% confidence intervals in brackets

^*^ *p* < 0.05, ^**^ *p* < 0.01, ^***^ *p* < 0.001

Thus, 10% higher GDP per capita yields -0.72% percentage reduction in infant mortality rates. In fact, this approach is very similar to our current framework. As shown the R-squared value 0.1301 and t-statistic 4.92 is exactly the same as in our current table 2. This is unsurprising because the correlation between these two forms of the dependent variable is basically 1.

To develop the offset, we could transform the -0.72% reduction in infant mortality to the unmet MDG progress measure that we adopted in our equation 1. This is a property of linear regression. If we transform the outcome variable by 100 * y, similarly our β coefficient transforms by 100 * β. Now, we could take this same transformation to the β-coefficient by similarly dividing it by 0.4 (i.e., actual change/target change), or 0.72/0/.4, yielding 1.798%, the same estimate as reported in the text.

Thus, we prove that the linear transformation of the dependent variable does not affect the outcomes presented.

References

1. Acock A (2008) A gentle introduction to STATA. College Station, Text, STATA Press.

<http://books.google.co.uk/books?id=YZIQO0acuWwC&dq=STATA+variance+inflation>.
