# Supplementary material for: Drivers of Inequality in Millennium Development Goal Progress: A Statistical Analysis
Source: PLoS Med. 2010 Mar 2;7(3):e1000241. doi: 10.1371/journal.pmed.1000241 (PMC2830449; doi:10.1371/journal.pmed.1000241)
Supplement: Text S6 — More details on the relationship between chronic NCDs and health MDGs. (0.05 MB DOC) [file pmed.1000241.s006.doc]

**Text S6. More details on the relationship between chronic NCDs and MDGs.**

High and increasing background rates of chronic NCDs appear to worsen the critical infectious disease and child health outcomes reflected in the MDGs: first, through household spending on long-term NCD care, which appears to be displacing resources for controlling health MDGs, and second, through direct biological pathways among those facing co-morbid NCDs and infectious diseases.

Tobacco and diabetes have also been strongly linked to worse tuberculosis outcomes. Extensive research has shown that smoking increases the risk of death from tuberculosis by at least two-fold.[1] Because more than one-in-five persons smoke in low-income countries, tobacco is a leading population attributable risk factor for tuberculosis (see appendix figure).[2,3,4] A meta-analysis of 13 observational studies found diabetes increasing the risk of developing tuberculosis by 3-fold.[5] In Mexico, one study attributed as much as one-quarter of pulmonary tuberculosis incidence in Mexico to diabetes, and a study by Dye and colleagues at the World Health Organization found that higher diabetes prevalence in urban areas was associated with a 15.2% highest smear-positive tuberculosis incidence in urban than rural settings.[6]

Thus, it is plausible that the high risks of these chronic NCDs among adults in poor households may contribute to the inability of families to address child mortality and place household members at higher risk for poor infectious disease outcomes, especially tuberculosis.

Table A. Selected Effects of Adult Health and Preventable Chronic Diseases & Injuries on MDG Progress, Non-Health Specific Goals

| Millennium Development Goal | Effects of Adult Health/Chronic Diseases |
| --- | --- |
| Goal #1. Eradicate extreme poverty and hunger | Paying for chronic disease care is a leading cause of bankruptcy in LMICs. |
| Goal #2. Achieve universal primary education | Overweight and obesity, which are more prevalent than malnutrition in LMICs, are associated with significant cognitive impairments and lower IQ scores among children.  Sick head-of-households reduce the probability that young children will attend primary school, as children instead spend time working or caring for sick parents. |
| Goal #3. Promote gender equality and empower women | Women traditionally assume care-taking duties for sick family members, which prevents them from participating equally in the labour market |
| Goal #7. Ensure environmental sustainability | Demand for and consumption of saturated fats and red meats is a leading contributor to climate change (methane from agribusiness). |
| Goal #8. Develop a global partnership for development | Addressing tobacco is regarded a ‘non-tariff barrier’ to trade. Without adequate formal safeguards for adult health, some countries are left with little choice but protectionism, as seen during the recent food crisis. |

**Note on joint HIV and NCD management**

Managing chronic infectious and chronic non-communicable diseases jointly has long been accepted in high-income countries. For example, both HIV/AIDS and NCDs have chronic and debilitating clinical manifestations, and result in long-term disability. Both require systems of long-term care and management, with patients playing greater roles in the success of treatment. Joint approaches to joint epidemics have a long history in rich countries. Before the 1950’s specialized clinics were established for dual treatment of ‘tuberculous diabetes’ in the UK.[7] Yet this is extremely important in countries undergoing epidemiologic transition in the developing world. HIV, a potentially chronic infectious disease if treated, shares important overlap with CVD and diabetes, and it has been suggested that the key logistical routes used to treat HIV and tuberculosis (such as directly observed therapy) can be used to further support prevention and care for chronic non-communicable diseases. Some reports now suggest that in India the lack of access to insulin is making diabetes a growing cause of death among HIV patients who are able to access anti-retrovirals. Yet learning and capacity building is only starting to take place now; new initiatives should seek to find common logistical approaches to these diseases rather than maintaining older dichotomies that fail to recognize their common presence in poor households.

References

1. Liu B, Peto, R, Chen, ZM, Boreham, J, Wu, YP, Li, JY, Campbell, TC, Chen, JS (1998) Emerging tobacco hazards in China: Retrospective proportional mortality study of one million deaths. British Medical Journal 317: 1411-1422.

2. Jha P, Jacob, B, Gajalakshmi, V, Gupta, PC, Dhingra, N, et al (2008) A nationally representative case-control study of smoking and death in India. N Engl J Med 358: 1137-1147.

1. Gajalakshmi V, Peto, R, Kanaka, T, Jha, P (2003) Smoking and mortality from tuberculosis and other diseases in India: retrospective study of 43000 adult male deaths and 35000 controls. The Lancet 16: 507-515.
2. Sitas F, Urban, M, Bradshaw, D, Kielkowski, D, Bah,S, Peto, R (2004) Tobacco attributable deaths in South Africa. Tobacco Control 13: 396-399.
3. Jeon C, Murray, MB (2008) Diabetes mellitus increases the risk of active tuberculosis: a systematic review of 13 observational studies. PLOS Med 5: e152.
4. Stevenson C, Forouhi, NF, Roglic, G, Williams, BG, Lauer, JA, Dye, C, Unwin, N (2007) Diabetes and tuberculosis: The impact of the diabetes epidemic on tuberculosis incidence. BioMed Central Public Health 7: 234.
5. Luntz G (1954) Tuberculous diabetics: the Birmingham Regional Service. Lancet 266: 973-974.
