# Supplementary material for: Drivers of Inequality in Millennium Development Goal Progress: A Statistical Analysis
Source: PLoS Med. 2010 Mar 2;7(3):e1000241. doi: 10.1371/journal.pmed.1000241 (PMC2830449; doi:10.1371/journal.pmed.1000241)
Supplement: Text S7 — Surveillance robustness check. (0.06 MB DOC) [file pmed.1000241.s007.doc]

**Text S7. Surveillance Robustness Check**

Robustness Check #1: Controlling for Surveillance Strength

Codifying WHO GBD surveillance levels (1a strongest surveillance to 4b weakest surveillance)

1a = 1.0

1b = 1.5

2a = 2.0

2b = 2.5

3a = 3.0

3b = 3.5

4a = none coded

4b = 4.5

Definitions:[1]

Levels 1 and 2: Death registration data, complete or incomplete, containing useable information on causes of death is available for the country, and used to adjust regional YLD distributions for causes with significant case fatality. Partial country-specific information on incidence or prevalence of non-fatal causes available.

Level 3: Other forms of information on child and adult mortality or causes of death (eg. verbal autopsy) available. Country-specific information on mortality for specific causes available.Partial country-specific information on incidence or prevalence of non-fatal causes available.

Level 4: Country information on level of adult mortality not available and it was predicted from child mortality level OR cause of death information for most causes not available, and cause pattern predicted using cause-of-death models. Partial country-specific information on incidence or prevalence of non-fatal causes available.

Table A: Control for Surveillance Strength

|  | (1) | (2) | (3) | (4) |
| --- | --- | --- | --- | --- |
|  | Unmet Infant Mortality Progress | Unmet Child Mortality Progress | Unmet TB Progress | Unmet HIV Progress |
| 10% higher GDP per capita | 1.42 [-0.47 to 3.31] | 1.55 [-0.52 to 3.63] | -0.80 [-1.95 to 0.34] | 0.95 [-0.65 to 2.55] |
| 1% higher Health Spending as percentage of GDP | 3.32 [-2.25 to 8.89] | 3.60 [-2.51 to 9.71] | -0.89 [-4.37 to 2.58] | -3.07 [-7.28 to 1.14] |
| $10 higher Health Spending per capita (PPP) | -0.0011 [-0.11 to 0.11] | -0.0066 [-0.14 to 0.12] | 0.064 [-0.027 to 0.15] | -0.058 [-0.19 to 0.075] |
| 1 additional physician/10,000 pop. | -0.72 [-1.61 to 0.18] | -0.65 [-1.57 to 0.26] | 1.08^***^ [0.46 to 1.70] | -0.18 [-1.23 to 0.86] |
| 10% higher NCD Mortality Rates | 4.54^*^ [0.33 to 8.75] | 4.02 [-0.41 to 8.46] | 6.83^***^ [3.65 to 10.0] | 2.16 [-2.32 to 6.64] |
| Surveillance level (1 – strong to 4.5 weak) | 24.0^**^ [6.91 to 41.1] | 25.5^**^ [6.90 to 44.1] | 8.34 [-2.12 to 18.8] | -2.74 [-16.7 to 11.2] |
| Number of Countries | 163 | 163 | 164 | 131 |
| *R*^2^ | 0.302 | 0.256 | 0.319 | 0.052 |

*Notes:* Results presented from four separate regression models. Constant estimated but not reported. 95% confidence intervals in parentheses. Unmet MDG Progress is calculated in percentage terms as 100 * [1 – (Actual ∆MR/Expected ∆MR)]. Progress towards reducing infant mortality rates and child mortality rates reflects MDG Targets 4.1 and 4.2 and modeled using a linear standard regression model. Progress towards halting or reversing tuberculosis mortality rates reflects MDG Target 6.9, and modeled using a linear probability model. Progress towards halting or reversing HIV prevalence reflects MDG Target 6.1, and modeled using a linear probability model. Data are from UN Millennium Development Goals Indicators 2008 edition.

^*^ *p* < 0.05, ^**^ *p* < 0.01, ^***^ *p* < 0.001

Test for homogeneity of effect: χ^2^(1) = 3.68, p = 0.0551

Robustness Check #2: Excluding countries where adult mortality was derived from child mortality

Table B: Excluding countries with low surveillance

|  | (1) | (2) | (3) | (4) |
| --- | --- | --- | --- | --- |
|  | Unmet Infant Mortality Progress | Unmet Child Mortality Progress | Unmet TB Progress | Unmet HIV Progress |
| 10% higher GDP per capita | 1.64 [-0.13,3.41] | 1.79 [-0.020,3.60] | 0.28 [-1.06,1.62] | 1.89 [-0.13,3.92] |
| 1% higher Health Spending as percentage of GDP | 2.02 [-2.72,6.76] | 2.08 [-2.72,6.89] | -1.96 [-4.87,0.95] | -1.77 [-7.58,4.05] |
| $10 higher Health Spending per capita (PPP) | -0.097 [-0.20,0.0089] | -0.11^*^ [-0.22,-0.014] | 0.0036 [-0.089,0.096] | -0.094 [-0.24,0.055] |
| 1 additional physician/10,000 pop. | -0.74 [-1.60,0.13] | -0.58 [-1.44,0.27] | 1.48^***^ [0.87,2.10] | -0.27 [-1.29,0.75] |
| 10% higher NCD Mortality Rates | 4.17 [-0.10,8.45] | 3.21 [-1.05,7.47] | 7.84^***^ [5.18,10.5] | 3.36 [-1.39,8.12] |
| Number of Countries | 115 | 115 | 116 | 89 |
| *R*^2^ | 0.117 | 0.083 | 0.441 | 0.072 |

*Notes:* Results presented from four separate regression models. Constant estimated but not reported. 95% confidence intervals in parentheses. Unmet MDG Progress is calculated in percentage terms as 100 * [1 – (Actual ∆MR/Expected ∆MR)]. Progress towards reducing infant mortality rates and child mortality rates reflects MDG Targets 4.1 and 4.2 and modeled using a linear standard regression model. Progress towards halting or reversing tuberculosis mortality rates reflects MDG Target 6.9, and modeled using a linear probability model. Progress towards halting or reversing HIV prevalence reflects MDG Target 6.1, and modeled using a linear probability model. Data are from UN Millennium Development Goals Indicators 2008 edition.

^*^ *p* < 0.05, ^**^ *p* < 0.01, ^***^ *p* < 0.001

Test for homogeneity of effect: χ^2^(1) = 1.82, p = 0.1767

*Sample code*

/** Robustness check table**/

eststo: regress unmet_imr lag2gdp healthgdpv2 heexp_ppp physcap logncdmr surveillance

eststo:regress unmet_under5mr lag2gdp healthgdpv2 heexp_ppp physcap logncdmr surveillance

eststo:regress unmet_tb lag2gdp healthgdpv2 heexp_ppp physcap logncdmr surveillance

eststo:regress f.unmet_hiv lag2gdp healthgdpv2 heexp_ppp physcap logncdmr surveillance

/** Test for homogeneity of effect**/

regress unmet_imr lag2gdp healthgdpv2 heexp_ppp physcap logncdmr surveillance

est sto t1

regress unmet_imr lag2gdp healthgdpv2 heexp_ppp physcap logncdmr

est sto t2

suest t1 t2

test [t1_mean]logncdmr = [t2_mean]logncdmr

References

1. World Health Organization (2009) Mortality and burden of disease estimates for WHO Member States in 2004. Geneva: World Health Organization.
