# Supplementary material for: Drivers of Inequality in Millennium Development Goal Progress: A Statistical Analysis
Source: PLoS Med. 2010 Mar 2;7(3):e1000241. doi: 10.1371/journal.pmed.1000241 (PMC2830449; doi:10.1371/journal.pmed.1000241)
Supplement: Text S8 — Sample alternative box plots. (0.03 MB DOC) [file pmed.1000241.s008.doc]

**Text S8. Sample Alternative Box Plots**
