# Supplementary material for: Drivers of Inequality in Millennium Development Goal Progress: A Statistical Analysis
Source: PLoS Med. 2010 Mar 2;7(3):e1000241. doi: 10.1371/journal.pmed.1000241 (PMC2830449; doi:10.1371/journal.pmed.1000241)
Supplement: Text S9 — Sample predicted progress based on NCD and HIV rates. (0.03 MB DOC) [file pmed.1000241.s009.doc]

**Text S9. Sample Predicted Progress based on NCD and HIV Rates**

Notwithstanding the limitations to predicting progress from a model to which the country contributed data points, we can illustrate the use of the model with some examples. Thus, in 2005 Niger had an infant mortality rate of 150 per 1,000 live births, compared with 191 per 1,000 live births in 1990. This corresponds to a reduction of 21.5%. However, a reduction of 40% would have yielded a drop of 76.4 to 114.6 deaths per 1,000 live births. Compared with the MDG target, Niger had achieved 53.7% progress, or 46.3% unmet progress. Given that Niger had very high age-standardised NCD mortality rates of 1029/100,000 population (53% higher than the sample mean of 671 per 100,000), we would have expected this to slow down progress by about 33.5%. Its HIV prevalence rates were about the sample average. On the other hand, Lesotho had rising infant mortality rates of 81 per 1,000 in 1990 to 102 per 1,000 live births by 2005, resulting in only 164.8% unmet progress. Its NCD rates were lower than the sample average (580 per 100,000) but HIV was far above average 23.2% prevalence. By our estimates this 20% higher HIV would translate into 163% unmet progress, which is very similar to the actual rate. These estimates also apply to MDG focus initiative countries, such as Ethiopia. Infant mortality dropped from 122 to 80 over the 1990-2005 period, resulting in 13.9% unmet progress. Its NCD mortality rates were 858 per 100,000 about 28% above the sample mean. This would have predicted about 17.6% unmet progress. Ethiopia’s HIV rates were 2, about 0.2% lower than the sample mean, reducing unmet progress by about 1.7%. Thus our estimate based on NCD and HIV would have predicted 15.9% unmet progress, which falls within the confidence interval bounds.
